# Rhizosheath microbial community assembly of sympatric desert speargrasses is independent of the plant host

**DOI:** 10.1186/s40168-018-0597-y

**Published:** 2018-12-04

**Authors:** Ramona Marasco, María J. Mosqueira, Marco Fusi, Jean-Baptiste Ramond, Giuseppe Merlino, Jenny M. Booth, Gillian Maggs-Kölling, Don A. Cowan, Daniele Daffonchio

**Affiliations:** 1 King Abdullah University of Science and Technology (KAUST), Biological and Environmental Sciences and Engineering Division (BESE), Thuwal, 23955-6900 Saudi Arabia; 2 Department of Biochemistry, Genetics and Microbiology, Centre for Microbial Ecology and Genomics, University of Pretoria, Pretoria, South Africa; 3 Gobabeb Research and Training Centre, Walvis Bay, Namibia

**Keywords:** Rhizosheath-root system, Plant-microbe interactions, Speargrasses, Stochastic assembly, Holobiont, Desert environment, Microbiome

## Abstract

**Background:**

The rhizosheath-root system is an adaptive trait of sandy-desert speargrasses in response to unfavourable moisture and nutritional conditions. Under the deserts’ polyextreme conditions, plants interact with edaphic microorganisms that positively affect their fitness and resistance. However, the trophic simplicity and environmental harshness of desert ecosystems have previously been shown to strongly influence soil microbial community assembly. We hypothesize that sand-driven ecological filtering constrains the microbial recruitment processes in the speargrass rhizosheath-root niche, prevailing over the plant-induced selection.

**Methods:**

Bacterial and fungal communities from the rhizosheath-root compartments (endosphere root tissues, rhizosheath and rhizosphere) of three Namib Desert speargrass species (*Stipagrostis sabulicola*, *S. seelyae* and *Cladoraphis spinosa*) along with bulk sand have been studied to test our hypothesis. To minimize the variability determined by edaphic and climatic factors, plants living in a single dune were studied. We assessed the role of plant species vs the sandy substrate on the recruitment and selection, phylogenetic diversity and co-occurrence microbial networks of the rhizosheath-root system microbial communities.

**Results:**

Microorganisms associated with the speargrass rhizosheath-root system were recruited from the surrounding bulk sand population and were significantly enriched in the rhizosheath compartments (10^5^ and 10^4^ of bacterial 16S rRNA and fungal ITS copies per gram of sand to up to 10^8^ and 10^7^ copies per gram, respectively). Furthermore, each rhizosheath-root system compartment hosted a specific microbial community demonstrating strong niche-partitioning. The rhizosheath-root systems of the three speargrass species studied were dominated by desert-adapted *Actinobacteria* and *Alphaproteobacteria* (e.g. *Lechevalieria*, *Streptomyces* and *Microvirga*) as well as saprophytic *Ascomycota* fungi (e.g. *Curvularia*, *Aspergillus* and *Thielavia*). Our results clearly showed a random phylogenetic turnover of rhizosheath-root system associated microbial communities, independent of the plant species, where stochastic factors drive neutral assembly. Co-occurrence network analyses also indicated that the bacterial and fungal community members of the rhizosheath-root systems established a higher number of interactions than those in the barren bulk sand, suggesting that the former are more stable and functional than the latter.

**Conclusion:**

Our study demonstrates that the rhizosheath-root system microbial communities of desert dune speargrasses are stochastically assembled and host-independent. This finding supports the concept that the selection determined by the desert sand prevails over that imposed by the genotype of the different plant species.

**Electronic supplementary material:**

The online version of this article (10.1186/s40168-018-0597-y) contains supplementary material, which is available to authorized users.

## Introduction

Deserts are dynamic and heterogeneous habitats that cover approximately one third of the global land surface [1]. Besides aridity, hot deserts impose additional stresses to their indigenous flora and fauna, including oligotrophy, elevated daily temperatures and sun irradiation, high salinity, strong wind erosion and environmental physical instability [1, 2]. Consequently, deserts are characterized by a lower biodiversity than other productive ecosystems [3–6] with specific ecological niches occupied by adapted macro- and micro-organisms [7, 8]. Specialized desert plants (xerophyte species) have notably evolved both their aerial (stem and leaf) and subterranean (root system) organs to prevent water loss, improve water storage and optimize water and nutrient uptake [9, 10].

Desert speargrass species (of the *Poaceae* and *Haemodoraceae* families) grow in sandy/rocky desert soils and have developed a ‘rhizosheath-root system’ as a xerophytic adaptive-trait [9, 11]. The rhizosheath is defined as the portion of soil that physically adheres to the root system and which can encase the entire root system of certain plants [9, 12]. As the rhizosphere, it is strongly influenced by root rhizodeposition. However, the rhizosphere can extend beyond the boundaries of the rhizosheath as it is not necessarily physically attached to the root system [12]. Root hairs, fungal hyphae and adhesive agents, such as microbial- and plant-derived mucilage, are responsible for the aggregation of the sand particles in the rhizosheath system [12–14].

The overall beneficial effect of developing a rhizosheath-root system has been demonstrated by the observation of a positive correlation between rhizosheath mass and plant growth under salt-stressed conditions [15]. In deserts, rhizosheaths have been also shown to provide mechanical protection to the root tissues, to promote water conservation and uptake under drought conditions and to positively influence nutrient uptake [11, 16]. Moreover, rhizosheaths represents a refuge and a resource for macro-organisms, as they can feed on the plant and live in relatively stable environmental conditions comparing to the fluctuating bulk desert sand habitats [9]. The rhizosheath structure provides also an ecological niche with favourable micro-climatic conditions, in which the higher water availability favours microbial growth and development, particularly of nitrogen-fixing bacteria [17, 18].

Despite the ecological services and protective advantages exerted by plant-associated microorganisms in deserts [3, 5, 19–23], few microbial cultivation-based studies have been conducted on the desert speargrass rhizosheath-root system [17, 18, 24, 25]. Such studies are also limited to a minor portion (the ~ 1% cultivable component) of microbial biodiversity and by their geographic range (mainly the Sinai desert) and the plant diversity (*Panicum turgidum, Stipagrostis scoparia, Bromus* spp., *Trisetaria koelerioides* and *Cyperus* spp., [17, 24, 25].

In order to evaluate the root microbiome recruitment strategies employed by desert speargrasses, we studied the root, rhizosheath and rhizosphere bacterial and fungal communities of three endemic-perennial Namib Desert speargrass species (*Stipagrostis sabulicola*, *Stipagrostis seelyae* and *Cladoraphis spinosa*) with a combination of scanning electron microscopy and molecular microbial ecology tools (qPCR and meta-barcoding). These selected plant species colonized the slope of a single dune in the central Namib Desert [26], allowing us to minimize the interference of factors such as biogeography, climatic or edaphic characteristics that could affect environmental microbial communities [27]. By exploiting this unique environmental setting, we aimed to disentangle the relationships between microbial communities and rhizosheath-root systems and to address fundamental questions on the recruitment strategies in the plant rhizosheaths.

The assembly of microbial communities in the plant rhizospheric zone is predominantly driven by the plant type in the natural ecosystem [27, 28]. However, due to the extreme environmental conditions in deserts, which drive a strong deterministic process of selection [29, 30], desert soil microbial diversity is reduced (i.e., lower biomass and richness) when compared to more productive ecosystems or arid soils under desert-farming management [3–6]. Consequently, we hypothesize that, in the Namib Desert dunes, the impact of plant species on the recruitment of their associated root system microbiota from the surrounding sandy soils would be minimal and thus that stochasticity would be a dominant driver [30, 31]. Nevertheless, we also expect niche-partitioning to play a role in the assembly process, and to detect a subset of phylogenetically consistent microbial groups (i.e., plant growth-promoting [PGP] microorganisms) that are ‘plant-species’- and/or ‘rhizosheath-root system compartment’-specific [32].

## Results

### Namib Desert speargrasses’ rhizosheath-root system structure

**Fig. 1:**
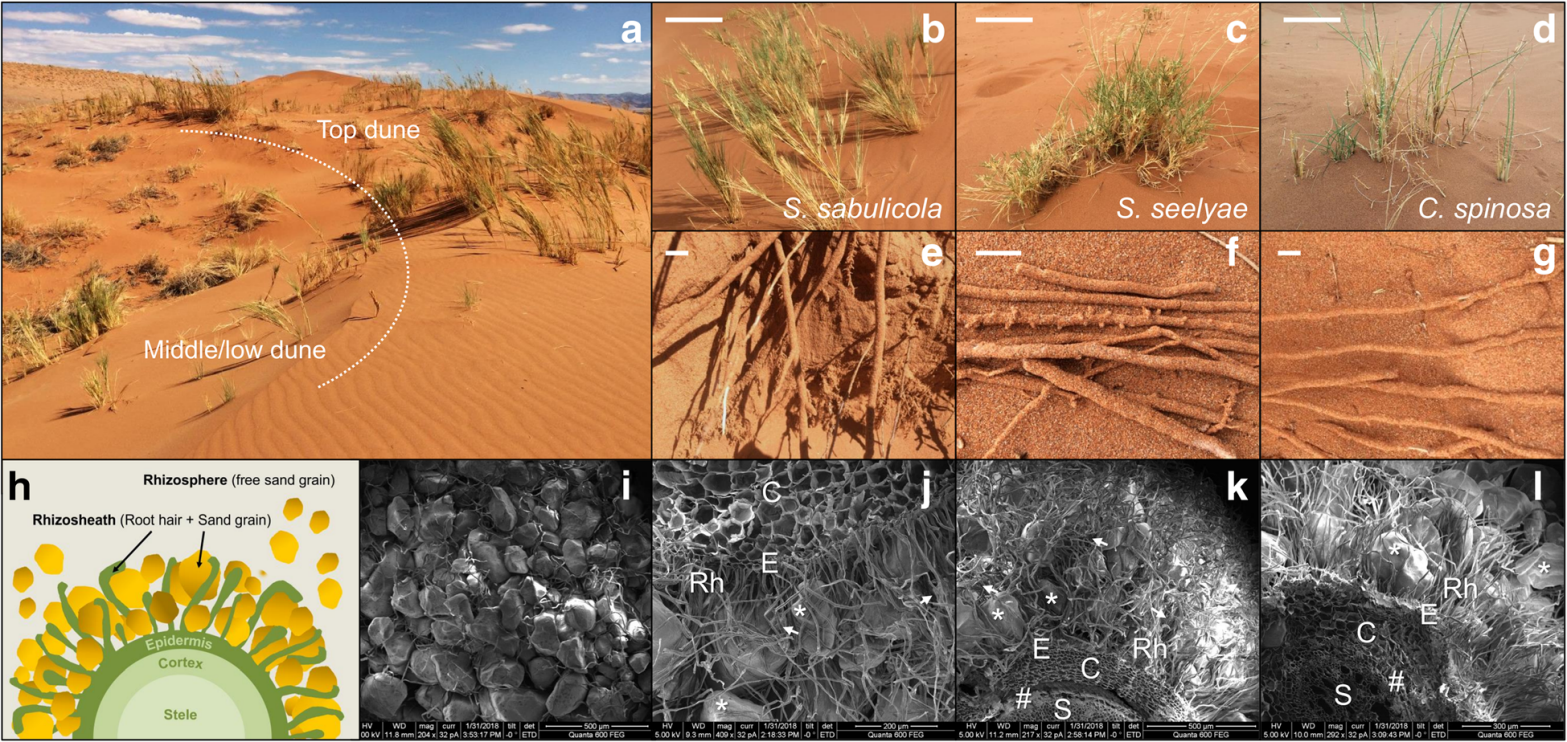
Habitat niches and rhizosheath-root systems of Namib Desert dune speargrasses. **a** Photograph of the sand dune selected for the sampling. Speargrasses’ habitat niches along dune slope were indicated (top vs middle/low dune; [26]). **b**–**d** Photographs of the three speargrasses (**b** *S. sabulicola*; **c** *S. seelyae* and **d** *C. spinosa*; bars correspond to 50 cm) and their respective rhizosheath-root system (**e**–**g** bars correspond to 1 cm). **h** Schematic representation of rhizosheath-root system structure. Root tissues composed by inner stele, followed by cortex and epidermal layers; rhizosheath composed by sand grains physically attached to the epidermal layer by the trapping effect of root hairs; rhizosphere referred to the sand grain influenced by root but not physically associated to the root system (sensu [12]). **i** SEM images of rhizosheath-root external layer showing rhizosheath matrix of root hairs entrapping sand grains. **j**–**l** Cross section SEM images revealing the structure of the speargrasses rhizosheath-root system (**j** *S. sabulicola*; **k** *S. seelyae* and **l** *C. spinosa*). S, stele: the central core of root of vascular plants; E, epidermis: the outermost cells of the root; C, cortical tissue: the tissue between the epidermis and the stele in root; Rh, root hairs: projection from the epidermis cells; *: sand grain surface; white arrow: mucilaginous, extra polysaccharide, fungal hyphae. #, endodermis: layer of cells between stele and cortical tissues. Note the different scales on the SEM photographs

The speargrasses studied were located on different slope sections of a single ~ 6 m high dune (Fig. 1a, chemical analysis Additional file 1: Table S1): *S. sabulicola* (Fig. 1b) occupied the middle/upper part of the dune slope (4.5 ± 0.15 m linear distance from the bottom of the slope), while both *S. seelyae* (Fig. 1c) and *C. spinosa* (Fig. 1d) grew on the lowest section of the dune slope (1.71 ± 0.17 and 1.4 ± 0.18 m, respectively). All three speargrass species showed a root system with a rhizosheath structure (Fig. 1e–g; schematic representation in Fig. 1h). The rhizosheaths appeared as thick and compact sandy cylinders covering the entire length of all roots, with an external layer composed of sand grains and root hairs (Fig. 1i). No significant differences between the rhizosheath diameters of the three speargrasses, defined as the sand physically attached to the root system (Fig. 1h; [12]), were observed (*F*_2,27_ = 0.83, *p* = 0.44). In contrast, mean root diameters differed significantly with plant species (*F*_2,27_ = 23.80, *p* < 0.0001): the largest for to *S. sabulicola* and the smallest for *C. spinosa* (Additional file 1: Table S2).

**Fig. 2:**
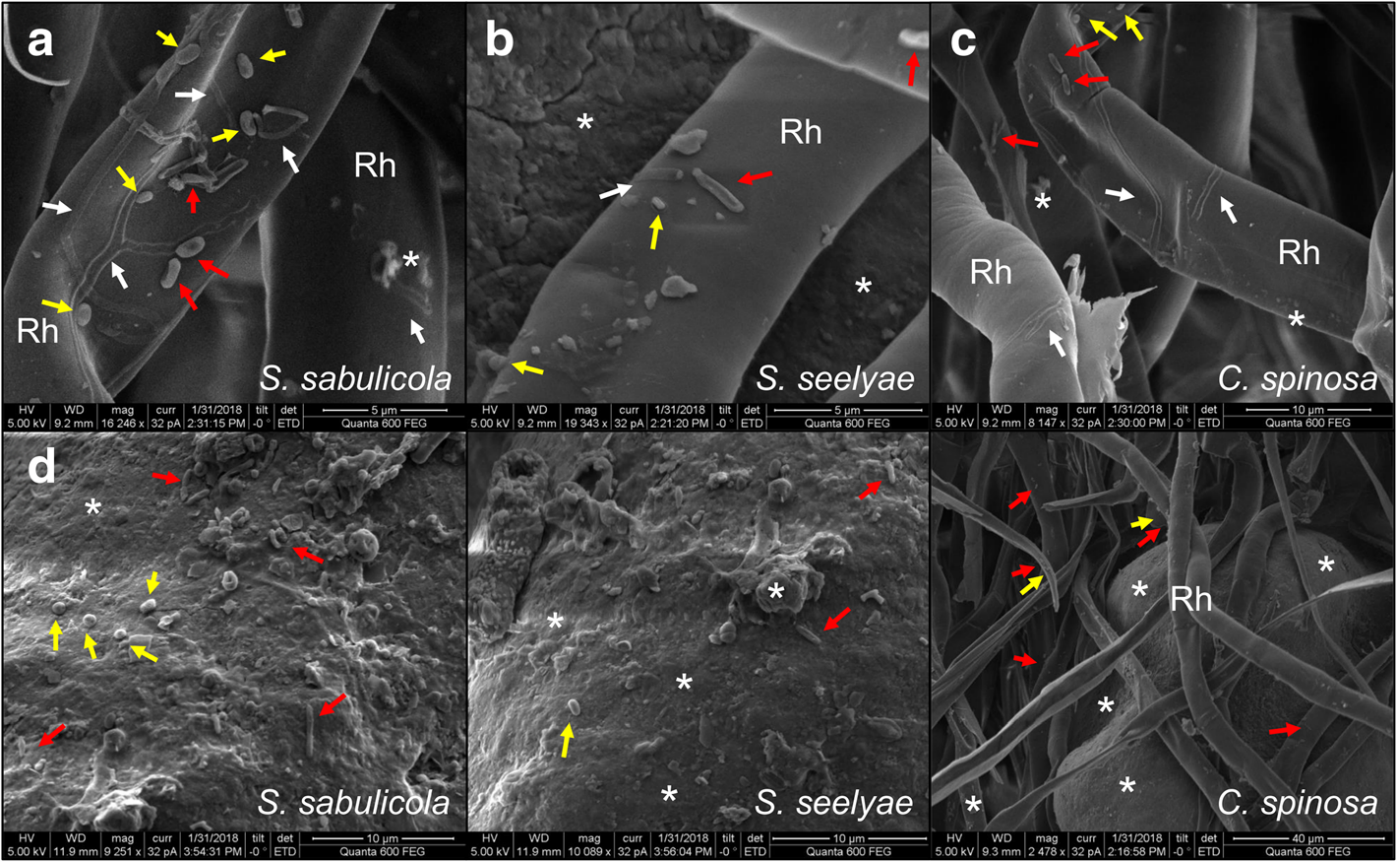
Visualization of microorganisms associated to the rhizosheath-root system of speargrasses. SEM micrograph showing bacterial cells present at the surface of both root hairs (**a**–**c**) and sand grains (**d**–**f**). Rh, root hairs: projection from the epidermis cells; *: flaky or coating materials; red-arrow: coccus-shaped bacteria; yellow-arrow: rod-shape bacteria; white-arrow: filamentous bacteria or fungal hyphae. Note the different scales on the SEM photographs

High magnification cross sections of intact rhizosheaths revealed the complex structure of this system (Fig. 1j–l), consisting of numerous long root hairs tightly binding fine and very fine sand particles and forming stable packaged arrangements (Fig. 1h, j–l). The surfaces of root hairs and sand grains showed flaky surface materials (stars in Fig. 2), possibly composed of mucilage and exopolymers released from roots and/or microorganisms [13, 14]. Magnified micrographs indicated the presence of microbial cells of different morphologies (including rod-shaped, coccus-shaped and filamentous bacteria, together with fungal hyphae) colonizing both the root hairs and the surfaces of sand particles (Fig. 2).

### Niche partitioning in speargrass rhizosheath-root systems

**Fig. 3:**
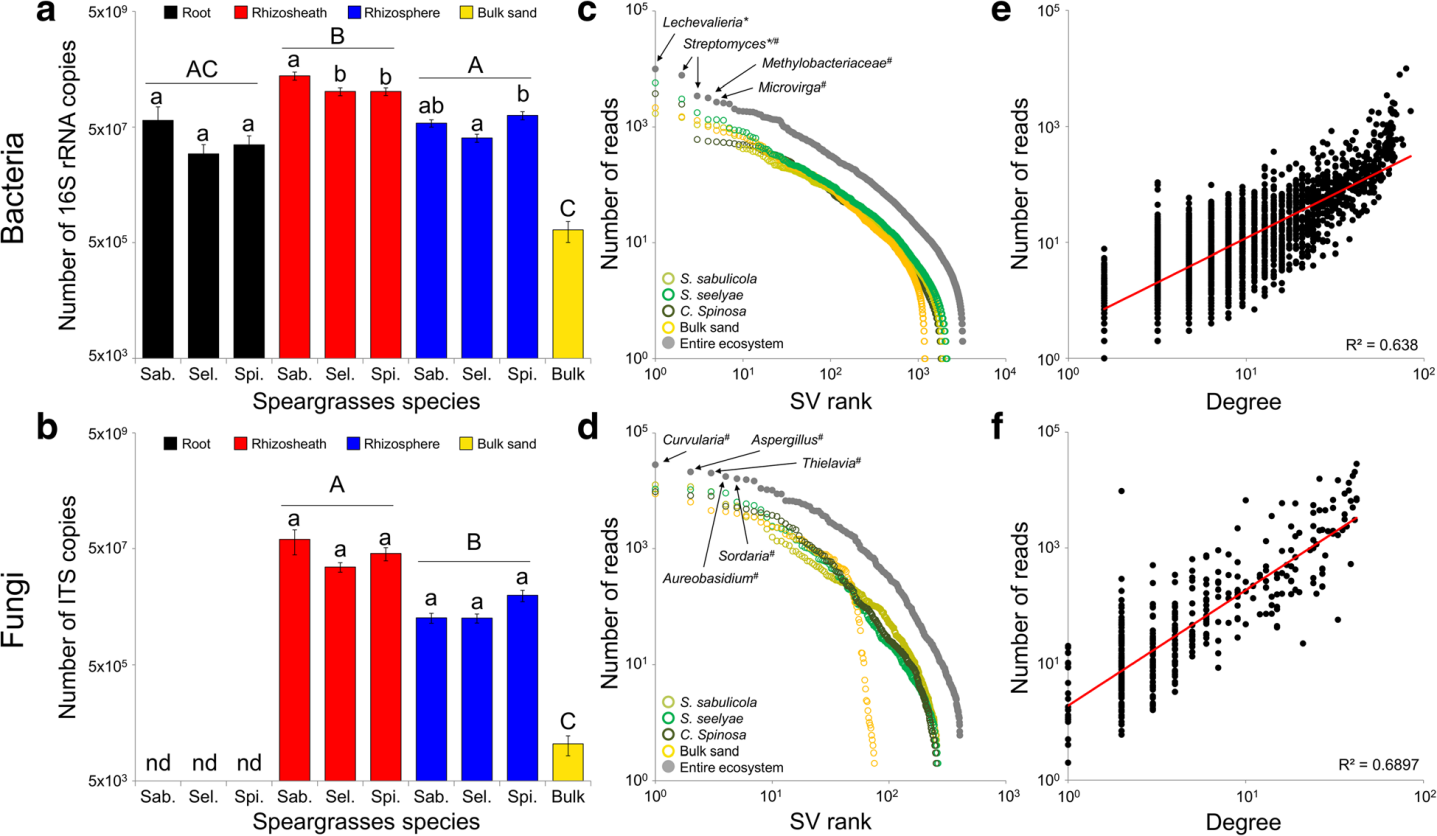
Speargrasses’ rhizosheath-root system microbial community distribution and composition. **a**, **b** Abundance of bacterial and fungal components in rhizosheath-root system (root tissues, rhizosheath, rhizosphere and bulk sand) of speargrasses measured by quantitative PCR of 16S rRNA and ITS gene copies per gram of sample, respectively. Lowercase letters indicated the significative difference (post hoc Dunn multiple comparison test) among speargrasses species for each compartment, while the significant difference among compartments was indicated by capital letters (post hoc Dunn multiple comparison test for bacteria and Mann-Whitney *t* test for fungi). n.d.: not detected. (**c**, **d**) Rank abundance distribution of bacterial (**c**) and fungal (**d**) SVs associated with speargrasses’ rhizosheath-root system (root, rhizosheath and rhizosphere) and bulk sand. (**e**, **f**) Power-law relationship between prevalence in speargrass rhizosheath-root system (measured by degree) and abundance (measured by the number of reads) for host-associated bacterial (**e**) and fungal (**f**) SVs. * not detected in bulk sand; # not detected in the internal root tissues

Bacteria were ubiquitously detected in the entire rhizosheath-root system, while fungi were not found in any root interior tissue. Quantification of copies of the bacterial 16S rRNA gene and of the fungal 18S–28S ribosomal internal transcribed spacers (ITS) suggested a progressive enrichment of the bacterial and fungal marker genes from the bulk sand (8.3 ± 3.3 × 10^5^ and 2.2 ± 0.8 × 10^4^ of bacterial 16S rRNA gene and fungal ITS copies per gram of sand, respectively) to the rhizosphere (5.7 ± 0.7 × 10^7^ and 4.8 ± 0.8 × 10^6^ copies per gram of rhizospheric sample), reaching the highest values in the rhizosheath (2.66 ± 0.3 × 10^8^ and 4.6 ± 1.2 × 10^7^ copies per gram of rhizosheath; multiple comparisons in Fig. 3a and b). There was a general dominance of bacteria (bacteria/fungi ratio: rhizosheath = 9 ± 1, rhizosphere = 15 ± 2 and bulk = 175 ± 117) at all sites. Inner root tissues showed lower bacterial 16S rRNA gene copy numbers (3.7 ± 1.7 × 10^7^), while fungal ITS sequences were non-detectable (Fig. 3a, b; [33]). Only the abundance of rhizosheath and rhizospheric 16S rRNA bacterial gene copies were differently affected by the plant host, with significantly higher values in *S. sabulicola* rhizosheath when compared to the other two species (Fig. 3a).

A total of 3224 bacterial 16S rRNA gene and 405 fungal ITS unique sequence variants (SVs) were identified globally in the rhizosheath-root system compartments and bulk sand samples (Table 1). Their distribution displayed a ‘dropping tail’ shape, with few abundant SVs and a large number of ‘rare’ SVs (bacterial and fungal reads abundances ranged from 2 to 99,585 and from 6 to 282,970, respectively; Fig. 3c, d). A significant relationship between microbial-occurrence in samples (degree) and microbial-abundance was detected, indicating a non-random SVs’ distribution across plant hosts (bacteria: adjusted *r*^2^ = 0.64, slope = 1.5, *p* < 0.0001; fungi: adjusted *r*^2^ = 0.69, slope = 2, *p* < 0.0001; Fig. 3e, f).

**Table 1:** Diversity estimates of microorganisms within each rhizosheath-root system compartment of the three speargrasses species

Microbe	Compartment	Plant species	N. sequence	Richness (N. SV)	Evenness (e^H/SV^)
Bacteria	Root	*S. sabulicola*	3336 ± 304	43 ± 6 (a)	0.416 ± 0.056 (a)
		*S. seelyae*	3760 ± 481	47 ± 5 (a)	0.472 ± 0.056 (a)
		*C. spinosa*	4526 ± 1031	29 ± 4 (a)	0.457 ± 0.059 (a)
		Total	3874 ± 603	40 ± 9 (A)	0.448 ± 0.029 (A)
	Rhizosheath	*S. sabulicola*	30,245 ± 1849	447 ± 12 (ab)	0.352 ± 0.032 (a)
		*S. seelyae*	33,743 ± 2521	491 ± 23 (a)	0.261 ± 0.022 (a)
		*C. spinosa*	27,905 ± 3998	391 ± 35 (b)	0.271 ± 0.026 (a)
		Total	30,631 ± 2938	443 ± 50 (B)	0.295 ± 0.05 (B)
	Rhizosphere	*S. sabulicola*	24,771 ± 1709	421 ± 18 (a)	0.375 ± 0.025 (a)
		*S. seelyae*	45,134 ± 3815	637 ± 26 (b)	0.279 ± 0.022 (b)
		*C. spinosa*	28,668 ± 3576	495 ± 49 (a)	0.373 ± 0.016 (a)
		Total	32,858 ± 10,809	518 ± 110 (C)	0.342 ± 0.055 (B)
	Bulk	Bulk	60,006 ± 4861	514 ± 42 (BC)	0.287 ± 0.013 (B)
Fungi	Rhizosheath	*S. sabulicola*	59,859 ± 7978	59 ± 6 (a)	0.156 ± 0.022 (a)
		*S. seelyae*	64,262 ± 5400	80 ± 4 (bc)	0.168 ± 0.018 (a)
		*C. spinosa*	67,420 ± 2531	71 ± 4 (ac)	0.196 ± 0.014 (a)
		Total	63,847 ± 3798	70 ± 11 (A)	0.173 ± 0.021 (A)
	Rhizosphere	*S. sabulicola*	64,100 ± 7471	88 ± 8 (a)	0.375 ± 0.025 (a)
		*S. seelyae*	83,517 ± 6803	68 ± 7 (a)	0.118 ± 0.018 (b)
		*C. spinosa*	74,045 ± 2396	86 ± 6 (a)	0.183 ± 0.031 (b)
		Total	73,887 ± 9709	81 ± 11 (A)	0.225 ± 0.134 (A)
	Bulk	Bulk	110,997 ± 26,291	20 ± 2 (B)	0.377 ± 0.06 (B)

Mean (± SD) of sequences, richness (number of SVs) and evenness were calculated for bacteria and fungi. Lowercase and uppercase letters in parenthesis indicated the results of post hoc multiple comparison (Tukey test) among plant species and rhizosheath-root system compartments, respectively. *SV* sequence variant, *H/SV* Shannon value divided by total number of sequence variant

**Fig. 4:**
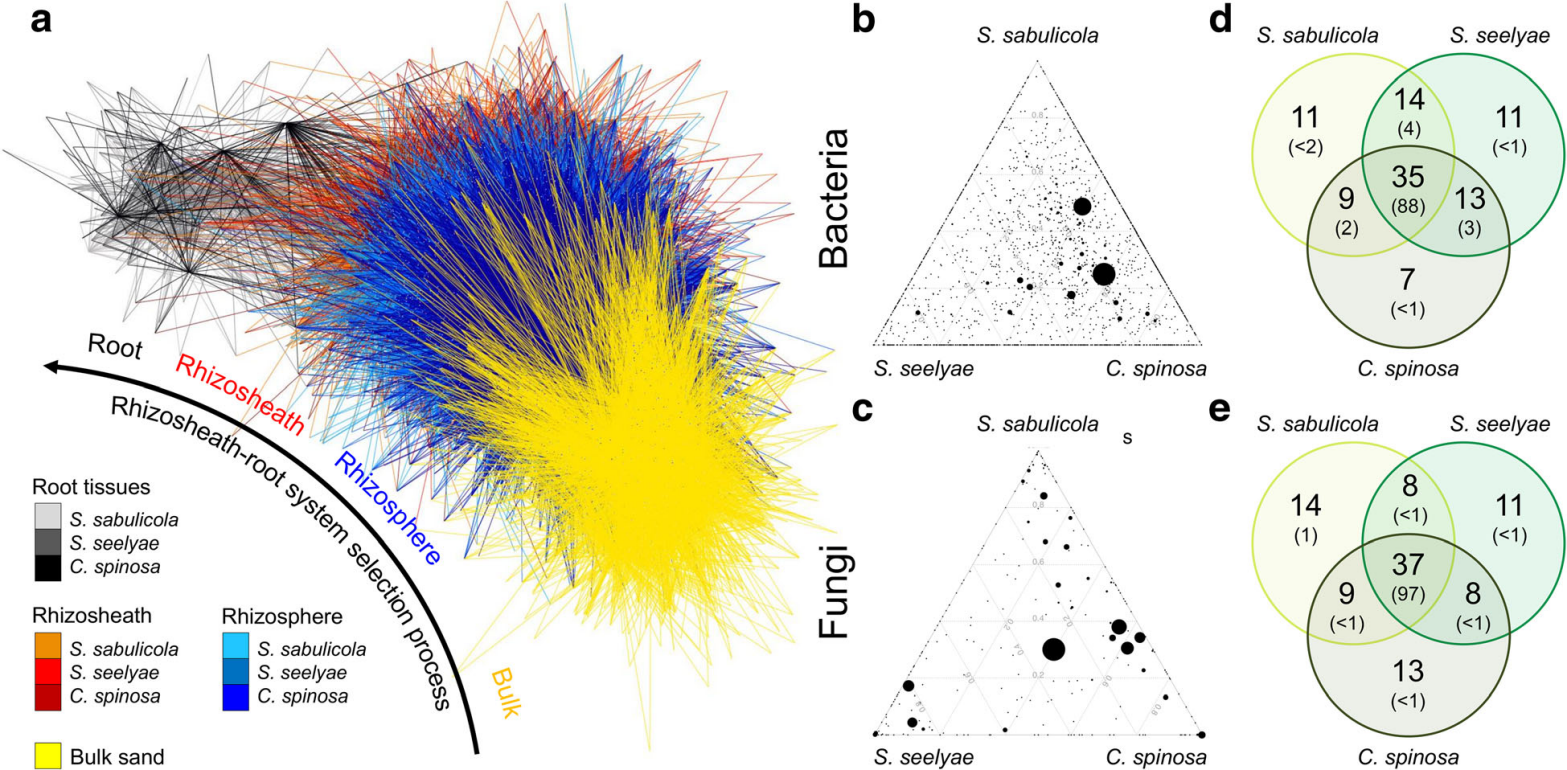
Namib Desert speargrasses rhizosheath-root systems recruitment process. **a** Bipartite network analysis of microbial communities (bacteria and fungi) associated with speargrass rhizosheath-root system and bulk sand. Edges connecting sample nodes to SV’ nodes were coloured according to their environmental source (black shade, root tissues; red shades, rhizosheath; blue shades, rhizosphere; yellow, sand soil). **b**, **c** Ternary plot revealing relative abundance (dot size) and generalist or plant species-specific behaviours of bacterial and fungal SVs among speargrasses. **d**, **e** Venn diagram detecting bacterial and fungal specialist (speargrass-specific) and generalist SVs (shared among speargrasses)

We found that the majority of the plant-associated bacterial and fungal SVs originated from the surrounding bulk sand (64 and 84%, respectively; Fig. 4a and Additional file 1: Figure S1). The bipartite network plot confirmed the selective process exerted by the rhizosheath-root systems and observed by the gene copy quantification: SVs present in the bulk sand were recruited by the rhizosheath and rhizosphere compartments and were then further filtered by the root rhizoplane barrier to finally become endophytic (Fig. 4a). Furthermore, each rhizosheath-root system compartment was found to have specifically associated microbiomes. This is further highlighted by the fact that only ~ 2% of the SVs were ubiquitously detected; i.e., associated to all the compartments of the rhizosheath-root system (Additional file 1: Figure S1 and S2). However, when excluding root tissues from the analysis, 35%–39% of bacterial SVs were found shared between the rhizosheath and the rhizosphere in the three species. Similarly, 54–59% of fungal SVs were shared between these two compartments. Altogether, and as no fungal SVs were detected in the plant roots, this confirmed that roots represent a strong filter in the process of plant root colonization by microorganisms [32].

Interestingly, when comparing the rhizosheath-root system microbiomes of the three speargrass species, ternary plots showed that most of the SVs had a generalist distribution (represented by the spheres in the middle of the triangles) instead of a host-specific distribution (spheres in the summit or along the edges of the triangles; Fig. 4b, c), with 35% of bacterial SVs and 37% of fungal SVs shared among all three plants. This represented 88% and 97%, respectively, of the total community (Fig. 4d, e). These values were conserved along the rhizosheath-root system compartments (Additional file 1: Figure S3).

### Drivers of microbial diversity in speargrasses’ rhizosheath-root systems

**Fig. 5:**
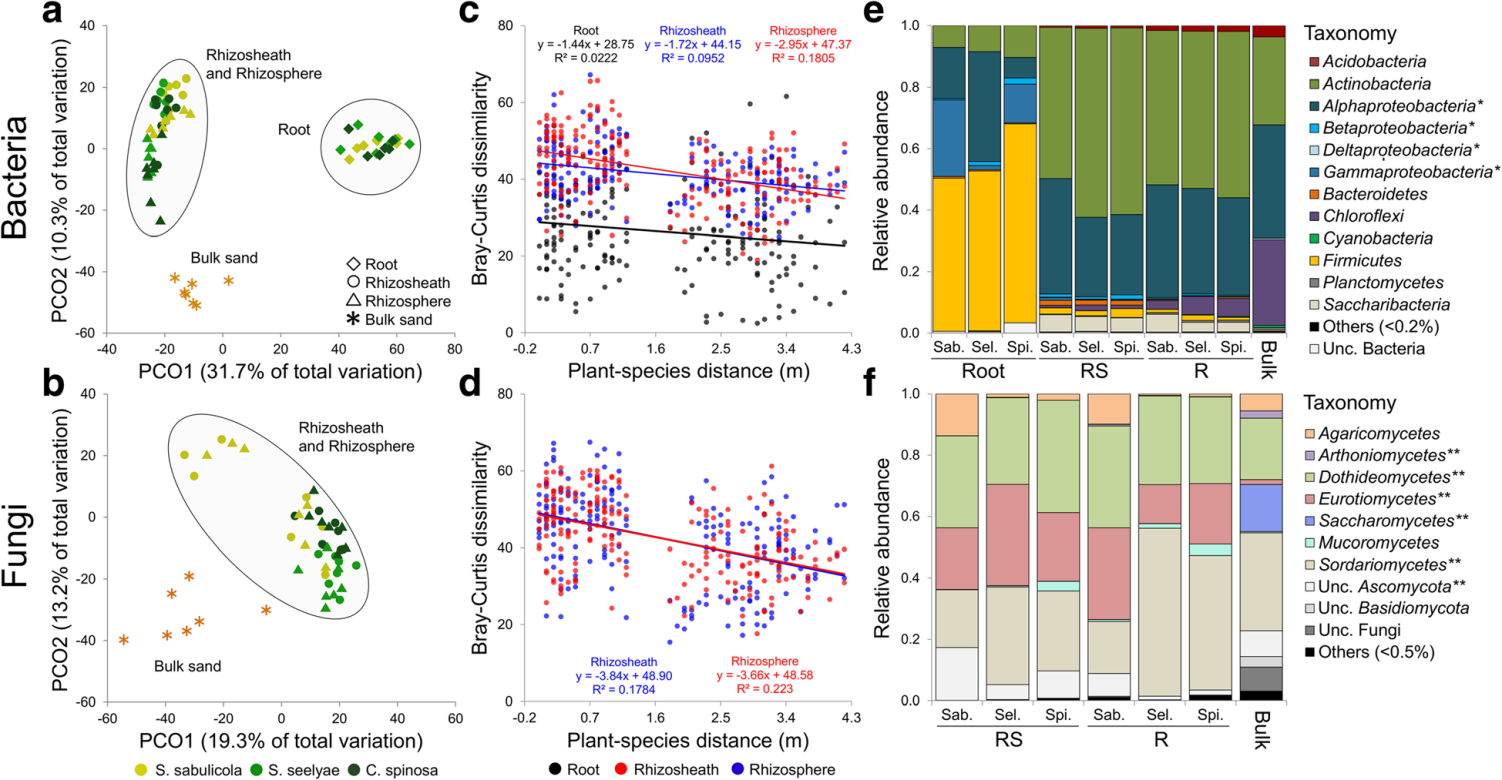
Diversity and taxonomical composition of microbial communities associated with the speargrasses rhizosheath-root system. **a**, **b** Principal coordinate analysis (PCoA) of **a** bacterial and **b** fungal communities associated with the rhizosheath-root system (root, rhizosheath and rhizosphere) and bulk sand. **c**, **d** Distance decay relationships of the communities’ dissimilarities (Bray-Curtis) of the bacterial (**c**) and fungal (**d**) components in the rhizosheath-root system compartments. **e**, **f** Relative abundance of bacterial (**e**) and fungal (**f**) phyla/classes associated with host and bulk sand. Relative abundance was expressed as percentage. One star (*) indicates classes belonging to *Proteobacteria* phylum; two stars (**) indicate classes belonging to *Ascomycota* phylum

A global segregation between the microbial community associated to rhizosheath-root system (host) and bulk sand was observed (PERMANOVA, bacteria: *F*_1,67_ = 6.11, *p* = 0.001; fungi: *F*_1,47_ = 8.06, *p* = 0.001), explaining up to 42% and 34% of the total compositional (Bray-Curtis) variation of bacterial and fungal taxa, respectively (Fig. 5a, b). Furthermore, for each plant species, a relatively high variability in rhizosheath-root system composition was observed (distance from centroid: bacteria 0.2–0.8 and fungi 0.4–0.8) compared to the surrounding bulk sand (bacteria 0.2–0.4 and fungi 0.3–0.5; Additional file 1: Table S3). However, no differences in the variability of plant species-associated microbial communities were observed (bacteria and fungi: *p* > 0.05; Additional file 1: Table S3).

More specifically, bacterial assemblages were significantly driven by the interaction of plant species and rhizosheath-root system compartments (bacteria: *F*_4,57_ = 2.078, *p* = 0.001; fungi: *F*_2,39_ = 0.740, *p* = 0.84), with the compartments being the major contributor (43%, Additional file 1: Table S4a; multiple comparisons in Additional file 1: Table S4b). For the fungal assemblages, the assembly was mainly driven by plant species (*F*_2,39_ = 6.211, *p* = 0.001; estimates of components of variation, 26%, Additional file 1: Table S4a; multiple comparisons in Additional file 1: Table S4c).

Mantel test results revealed a significant correlation (*p* < 0.05 in Additional file 1: Table S4d) between the compositional variation of bacterial and fungal rhizosheath and rhizosphere beta-diversity values and distance from the dune base (habitat-niche). This was not observed for the root bacterial communities (Additional file 1: Table S4d). Furthermore, a significant decline in compositional similarities with linear distance was also found for the rhizosheath and rhizosphere sandy compartments (Fig. 5c, d; Additional file 1: Table S5). Altogether, these results show that the closer the proximity of the individual plants, the more similar their rhizosheathic and rhizospheric communities were, and vice versa; i.e., that communities from *S. seelyae* and *C. spinosa* that are localized on the middle/low section of the dune hosted similar communities, when compared to those of *S. sabulicola* which grew near the dune top. In contrast, the root endophytic bacterial communities were not significantly influenced by inter-plant distance (Fig. 5c and Additional file 1: Table S5), further suggesting the strong selection of the endophytic root microbiome by the speargrass, independent of their location and plant taxonomy.

### Assembly dynamics of bacterial and fungal communities associated with the speargrass rhizosheath-root system and bulk sand

Bacterial and fungal communities were characterized by different alpha-diversity (richness and evenness) trends (Table 1). While bacterial communities hosted by sandy-compartments of rhizosheath system (rhizosheath and rhizosphere) showed similar alpha-diversity values compare to bulk sand, the fungal alpha diversity values were significantly higher than those of the bulk sand (multiple comparisons in Table 1). The endophytic root bacterial communities were always significantly less diverse (low richness) and more equal (high evenness) than all the sand-dominated samples (rhizosheath, rhizosphere and bulk sand). Furthermore, speargrass species did not influence root tissue bacterial alpha diversity but affected the richness and evenness of rhizosphere microbial communities (Table 1). *S. seelyae* presented the lowest bacterial evenness and the highest bacterial richness, and *S. sabulicola* exhibited the highest fungal evenness. In the rhizosheath, plant species only influenced the richness values, with variable effects for bacterial and fungal communities (Table 1).

Phylogenetic diversity was measured using different metrics: phylogenetic distance between SVs (PD/SV), nearest taxon index (NTI) and mean relatedness index (NRI). The PD/SV ratios of the rhizosheath-root system bacterial communities did not differ between plant species or with the bulk sand (*F*_3,64_ = 0.24, *p* = 0.9; Newman-Keuls multiple comparison *p* > 0.05; Table 2). This was also observed when considering each compartment individually (root: *F*_2,16_ = 0.489, *p* = 0.6; rhizosheath: *F*_2,18_ = 0.034, *p* = 0.1; rhizosphere: *F*_2,18_ = 1.9, *p* = 0.2). Conversely, fungal communities showed a significantly lower phylogenetic diversity in plant-influenced compartments when compared to the bulk sand (*F*_3,45_ = 341.8; *p* < 0.0001).

**Table 2:** By host comparison of phylogenetic diversity in speargrasses species and bulk sand

Microbe	Index	ANOVA	*S. sabulicola*	*S. seelyae*	*C. spinosa*	Bulk sand
Bacteria	PD	F_3,64_ = 0.24, *p* = 0.9	0.058 ± 0.013 (a)	0.055 ± 0.017 (a)	0.058 ± 0.019 (a)	0.054 ± 0.008 (a)
	NRI	F_3,64_ = 16.7, *p* < 0.0001	5.57 ± 2.63 (a)*	4.40 ± 1.71 (a)*	5.29 ± 2.33 (a)*	−0.99 ± 1.77 (b)
	NTI	F_3,64_ = 3.98, *p* = 0.015	4.69 ± 1.94 (a)*	4.32 ± 1.38 (a)*	4.84 ± 1.53 (a)*	2.42 ± 1.88 (b)
Fungi	PD	F_3,45_ = 342, *p* < 0.0001	0.29 ± 0.03 (a)	0.27 ± 0.03 (a)	0.27 ± 0.02 (a)	0.95 ± 0.12 (b)
	NRI	F_3,45_ = 3.7, *p* = 0.018	0.70 ± 0.76(a)	0.25 ± 1.28 (ab)	0.35 ± 0.80 (ab)	−0.79 ± 0.96 (b)
	NTI	F_3,45_ = 4.7, *p* = 0.0062	0.98 ± 0.55(ab)	1.35 ± 0.92 (a)	1.61 ± 0.91 (a)	0.26 ± 0.90 (b)

Faiths’ phylogenetic distance per SV (PD/SV), net relatedness index (NRI) and nearest taxon index (NTI) have been used as metrics to evaluate bacterial and fungal alpha phylogenetic diversities. Lowercase in parenthesis indicated the results of post hoc multiple comparison (Newman-Keuls test) among plant species

*Communities that are significantly structured at the *p* < 0.05 level

Bulk sand bacterial communities and all fungal communities were found to be randomly structured phylogenetically (*p* > 0.05; Table 2), while the rhizosheath-root system bacterial communities were significantly clustered for both NRI (1.6 < NRI < 11.2; *p* < 0.05) and NTI (0.9 < NTI < 8.9; *p* < 0.05) metrics without significant changes within the different plant species (Table 2). Moreover, the lack of correlation between sequencing depth and NRI (linear regression: bacteria, *p* = 0.08; fungi, *p* = 0.33) or NTI (linear regression: bacteria, *p* = 0.22; fungi, *p* = 0.69) suggested that the addition of infrequent taxa to the communities would not alter the phylogenetic structure. The low phylogenetic alpha diversity of speargrass-associated microbial communities and absence of any correlation between these communities and the position of the plants along the dune slope (Mantel test, Additional file 1: Table S6) indicated that the three speargrasses species hosted similar microbial communities (*p* > 0.05; Table 2).

The phylogenetic relatedness of the bacterial and fungal communities was analysed by calculating both the ‘basal’ and ‘terminal’ metrics of phylogenetic beta diversity (ßNRI and ßNTI, respectively), in order to evaluate the phylogenetic turnover [31]. Both ßNRI- and ßNTI-bacterial scores were between − 2 and + 2 (0.32 < ßNRI< 0.66; 0.02 < ßNTI< 0.44), which is consistent with random phylogenetic turnover; i.e., where stochastic and/or ecologically-neutral factors play important roles in community assembly, a process known as neutral community assembly [31]. Similarly, fungal communities showed a neutral rhizosheath-system community assembly, with both ßNRI and ßNTI values between − 2 and + 2 (1.11 < ßNRI< 1.59; 0.09 < ßNTI< 0.82). No correlation between the phylogenetic beta diversity metrics and spatial distance among speargrass rhizosheath habitat-niches was detected (Mantel test, *p* > 0.05), suggesting a consistent stochastic mechanism of assembly of the rhizosheath-root system microbial communities. This is most probably linked to the low biomass and richness/phylogenetic alpha diversity detected in the bulk sand communities, from which the rhizosheath-root system microbial communities are recruited (Fig. 3; Tables 1 and 2).

### Bacterial and fungal community taxonomic compositions and predictive functions in speargrass rhizosheath-root systems

The complete phylogenetic dataset comprised a total of 21 bacterial phyla (99.8% sequences classified), 51 classes (96% classified), 68 orders (93% classified), 114 families (88% classified) and 215 genera (72% classified; Additional file 1: Table S7a). The interaction of plant species and rhizosheath-root system compartments significantly influenced the distribution of bacterial taxa at the phylum/class and family levels (phylum/class: *F*_4,53_ = 2.09, *p* = 0.026; family: *F*_4,53_ = 2.65, *p* = 0.001; Additional file 1: Table S8a and b). Notably, the few abundant bacterial members (SVs), accounting for 15% relative abundance, were affiliated to *Actinobacteria* (5 and 6% to *Lechevalieria* and *Streptomyces*, respectively) and *Alphaproteobacteria* (1.5 and 1.7%, *Microvirga* [reclassification of *Balneimonas*] and *Methylobacteriaceae*, respectively; Fig. 3c). In particular, plant-associated rhizosheath and rhizosphere communities showed high abundances of *Actinobacteria* (49–62%) and *Alphaproteobacteria* (26–38%) while root tissues were mainly dominated by *Firmicutes* (50–65%), with *Gammaproteobacteria* (1–25%) and *Actinobacteria* (7–10%) in lower abundance. Bulk sand bacterial communities were mainly composed of *Alphaproteobacteria* (37%), *Actinobacteria* (29%) and *Chloroflexi* (28%; Fig. 5e, Additional file 1: Table S7a). At the family level, *Pseudonocardiaceae*, *Streptomycetaceae*, *Methylobacteriaceae*, *Nocardioidaceae*, *Hyphomicrobiaceae*, *Microbacteriaceae* and *Micrococcaceae* showed specific distributions in the rhizosheath and rhizosphere compartments, while *Bacillaceae*, *Rhizobiaceae* and *Pseudomonadaceae* were the dominant taxa in the root tissues, but with a host-specific distribution (Additional file 1: Table S9a). In fungal communities six phyla were detected (92% sequences classified), distributed across 22 classes (85% classified), 37 orders (84% classified), 57 families (73% classified) and 72 genera (70% classified; Additional file 1: Table S7b). The classes *Sordariomycetes*, *Eurotiomycetes and Dothideomycete* (all *Ascomycota*) equally dominated the datasets (Fig. 5f). The global fungal class distribution was significantly affected by plant species (*F*_2,39_ = 2.51, *p* = 0.027; Additional file 1: Table S8b). At a lower taxonomic rank (genus), fungal composition was significantly influenced by both plant species and rhizosheath-root system compartments (*F*_2,39_ = 5.73, *p* = 0.001 and *F*_1,40_ = 2.10, *p* = 0.008, respectively; Additional file 1: Table S8d), with 26% of the genera showing a different abundance in function of the plant species (for instance, *Fusarium* and *Volvopluteus*) and only one genus (*Cladosporium*) in function of compartments (Additional file 1: Table S9b). Among fungal genera, members belonging to the *Curvularia*, *Aspergillus*, *Thielavia*, *Aureobasidium* and *Sordaria* (all *Ascomycota*) showed high relative abundance (28% of all reads; Fig. 3d) with a generalist-distribution(FDR-*p* > 0.05) independent of plant species or compartments.

### Meta-network topology and microbial community interactions in the speargrass rhizosheath-root system

**Fig. 6:**
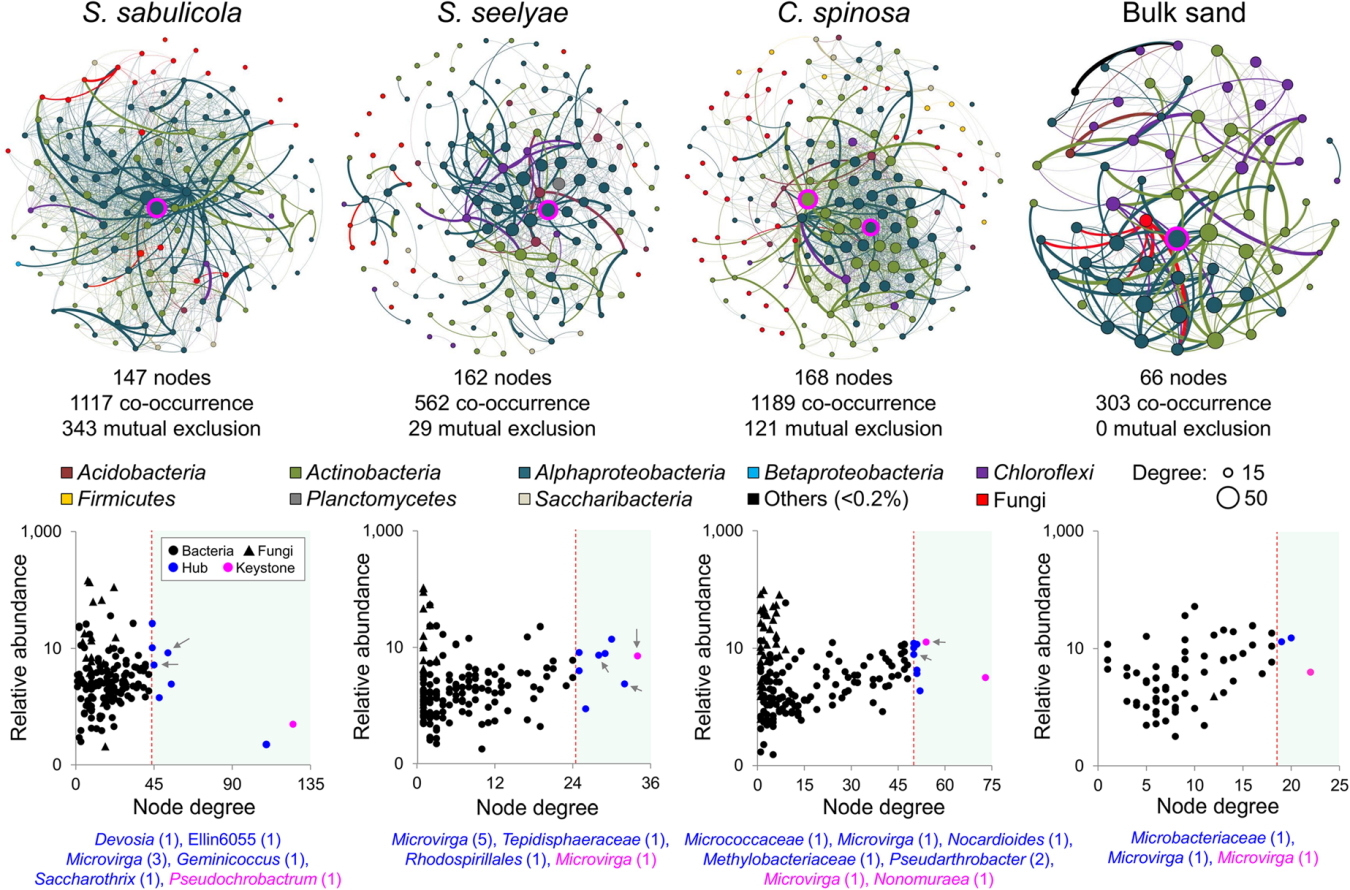
Co-occurrence of microbial SVs in speargrasses rhizosheath-root system and bulk sand. Significant interaction (co-occurrence and mutual exclusion) between bacteria and fungi SVs in *S. sabulicula*, *S. seelyae*, *C. spinosa*, and bulk sand were visualized by co-occurrence network (upper panels). Circles (nodes) represent SVs (bacteria and fungi) significantly interacting in the microbial networks. Size of circles indicates the degree of connection. Nodes (SVs) were colored according to their taxonomic affiliation. Edges were colored by the taxonomic affiliation of their origin-node. Relative abundance (as counts per million, CPM) of all SVs significantly interacting in speargrass and bulk sand co-occurrence networks was visualized as a function of their degree of co-occurrence (lower panels). In the lower panel, hub SVs were colored in blue and keystone SVs are displayed in pink. Arrows indicated hubs (blue dot) and keystone SVs (pink dot) belonging to the *Microvirga* genus detected in the three plant networks. Keystone SV nodes were also indicated with a pink border in the co-occurrence network images (upper panels)

A significantly higher number of microbial (bacterial and fungal) community members in the three plant rhizosheath-root systems (147 in *S. sabulicola*, 162 in *S. seelyae*, 168 in *C. spinosa*) than in the bulk sand (66) established significant and non-random interactions. Such pattern was observed for the numbers of co-occurrences as well (1117, 562, 1189 and 303, respectively; Fig. 6; Table 3); bulk soil showed only co-presence relationships, while speargrass rhizosheath-root systems showed both co-presence and mutual exclusion (Fig. 6). Furthermore, the microbial components (nodes) of the three speargrass networks showed significantly different degrees of connection (*F*_2,452_ = 58.01, *p* < 0.001; highest for *S. sabulicola*), closeness centrality (*F*_2,452_ = 107.52, *p* < 0.001; highest for *S. sabulicola*), betweenness centrality (*F*_2,452_ = 4.11, *p* < 0.05; highest for *S. seelyae*) and average shortest path length (*F*_2,452_ = 107.52, *p* < 0.001; highest for *S. seelyae*).

**Table 3:** Properties of microbial interaction (bacteria and fungi) in speargrasses’ rhizosheath-root systems and bulk sand co-occurrence networks

Community	^a^ SV		^b^ Connections			Hub/keystone		^c^ Connectivity
	Bacteria	Fungi	Bac-Bac	Fun-Fun	Bac-Fun	Bacteria	Fungi	Network-wide
*S. sabulicula*	126	21	1222	21	217	8/1	0	19.86
*S. seelyae*	147	15	571	2	18	8/1	0	7.30
*C. spinosa*	132	36	1220	16	74	9/2	0	15.60
Bulk sand	65	1	292	0	11	6/1	0	9.18

^a^Number of network nodes

^b^Number of network edges

^c^Mean number of connections per node (degree)

Each plant species had also a characteristic taxonomic profile with respect to central interactions (edge betweenness centrality: *F*_56,3259_ = 5.50, *p* < 0.001; Additional file 1: Figure S4). For *C. spinosa*, central interactions originated principally from *Actinobacteria* co-occurring with fungi, *Saccharibacteria* interacting with *Actinobacteria* and *Firmicutes* and *Basidiomycota* co-occurring with *Ascomycota* and *Actinobacteria*. For *S. seelyae,* dominant central interactions were between *Acidobacteria* and *Saccharibacteria*, and for *S. sabulicola*, between *Alphaproteobacteria* and *Betaproteobacteria*, albeit with lower values (Additional file 1: Table S10 and Additional file 1: Figure S4). SVs belonging to *Deltaproteobacteria*, *Gammaproteobacteria*, *Bacteroidetes*, *Cyanobacteria* and *Arthoniomycetes* (1.6 and 0.8% of the total bacterial and fungal reads, respectively) did not show significant co-occurrences with any other microbial community taxon.

Members with high degrees of co-occurrence (top 5%) were identified as hubs. All the detected hubs had medium/low relative abundance (as counts per million, CPM; Fig. 6) and were taxonomically affiliated to *Actinobacteria* and *Alphaproteobacteria*, with *Planctomycetes* in *S. seelyae* (Additional file 1: Table S10, see hub column in node tables). More hubs were detected in speargrass rhizosheath-root system networks (8, 8 and 9 hubs in *S. sabulicola*, *S. seelyae* and *C. spinosa*, respectively) when compared to the bulk sand (3 hubs; Additional file 1: Table S10). All these hubs established heterogeneous interactions with bacterial and/or fungal nodes, depending on the plant species (Additional file 1: Table S10, see edge tables)*.* Interestingly, only three hubs, belonging to the *Microvirga* genus (one of the most abundant taxa detected overall, Fig. 3a), were identified in all the plant-related networks, while the other hubs were plant- and bulk sand-specific.

Keystone taxa were defined as taxa interacting with many other members (i.e., top 1% of interactions); such taxa are thought to play crucial roles in the overall community [34]. The four meta-networks hosted a keystone species belonging to the *Alphaproteobacteria* class (genus *Pseudochrobactrum*in *S. sabulicola,* genus *Microvirga* in *S. seelyae, C. spinosa* and bulk sand; Additional file 1: Table S10, see keystone column in node tables). Only the *C. spinosa* network showed a second keystone species, affiliated to the *Actinobacteria* phylum (genus *Nonomuraea*; Fig. 6; Additional file 1: Table S10).

## Discussion

The complex moisture/sand mobility gradient along the slope of Namib Desert dunes determines specific micro-niches that strongly influence the species, number and distribution of perennial speargrasses [26, 35]. According to Yeaton [26], *S. sabulicola* is well adapted to grow on the upper dune slopes which are characterized by very mobile sand and higher moisture availability. With increasing sand stability and lower moisture; i.e., moving towards the dune base, others speargrasses, such as *S. seelyae* and *C. spinosa,* are typically found [9, 26, 35]. In the higher parts of the dunes, plants with strong root systems are more successful at establishing in moving sands [26], as indicated by the significantly wider diameter of the rhizosheath-root system (entire and single root internal tissues) of *S. sabulicola*. Beside the genetic predisposition for rhizosheath development, soil properties and soil texture/granulometry [36, 37] delineate the final shape and size of the rhizosheath. This explains the fact that rhizosheath width (sandy coating) of the three speargrasses growing in the same sandy substrate was similar.

In dry soils and xeric-stressed environments (e.g. desert and gravel plain soils) rhizodeposition occurs around and along the entire root length resulting, in some species, in the formation of a compact rhizosheath structure associated with plant stress tolerance [11]. Such rhizosheaths were always significantly enriched in microbial cells, even compared to the rhizosphere. The structure and composition of the rhizosheath, which includes the presence of exudates, mucigels and exopolymers, increases the wettability and water absorption capacity of the root system and generates a favourable microenvironment [16] for the establishment of highly diverse bacterial and fungal populations [14, 24, 25, 33]. The higher number of cells associated with the rhizosheath is supported by the microscopic observations of numerous bacteria and fungal hyphae associated with root hairs and sand grains [14, 33, 38]. Recruitment of microaggregates (< 250 μm, i.e., fine and very fine sand) by the rhizosheath compartment may also drive greater microbial diversity [39].

For recruitment, diversity and interactions of the microbiome components associated with the rhizosheath-root system of desert speargrasses, abiotic filtering (deterministic factors) imposed by the harsh conditions of the desert [30] reduces the microbial pool available in the surrounding bulk sand to a limited number of members sharing similar adaptive-traits [40], which may contribute to their adaptation and survival [6, 19, 41]. Through a process most probably mediated by the plant rhizodeposition, the rhizosheath-root system of speargrass selects its microbiome from the microbial pool present in the surrounding sand. An additional selection step, at the rhizoplane level, allows only certain bacteria to colonize the root tissues [32]. This selective process ultimately leads to a sequential differentiation within the successive compartments of the rhizosheath-root system of the three speargrass species, supporting the concept that root compartmentalization is the major driver of plant-microbe interaction in arid and semi-arid environments [19, 41]. Recent omics-based analyses have shown that plant seeds contain microbes that can be transmitted from one plant generation to the next and have profound impacts on plant ecology, health and productivity [42]. Seeds’ microbiome can be both vertically transmitted from plant tissues and horizontally transferred from the surrounding environment (i.e. sand); it represents the culmination of a complex process of microbial interactions mediated by plant throughout its life cycle [43]. In the rhizosphere of juvenile maize (21 days old) grown in both sterile and non-sterile substrates, identical dominant bacterial were observed, indicating seeds as a source of inoculum [44]. However, rhizospheres developed in non-sterile substrates harboured greater bacterial diversity than sterile ones, confirming that the surrounding soil remains very important in determining the assembly and structuring of rhizospheric communities [32, 45]. The observed filtering process mediated by the rhizosheath-root system suggests that a reduced number of bacteria, and possibly no fungi, can be vertically transferred to the speargrass seeds and that the surrounding environment (i.e. sand) represents the main source of microorganisms associated with such perennial plants.

In the rhizosheath-root structure, a homogeneous distribution of microorganisms among the three plant species was observed, defining a ‘core microbiome’, in which the microorganisms available in the sand colonize the different host species [46]. In addition, the prevalence of microbial generalists over specialists indicates an inter-species sharing of the rhizosheath-root system microbiota among speargrass, possibly as a consequence of a weak selection mediated by the plants due to stochastic factors. Such factors may include probabilistic processes that homogenize sand microbial communities, such as sand mobility and random changes in microbial species relative abundances (ecological drift [30, 31]). As frequently observed in soils, both deterministic and stochastic forces act on desert microbial populations [2, 7, 29, 30, 47]. This complex balance is mainly influenced by stochasticity [30, 48]. Abiotic filtering (i.e. the desert microenvironment) and biotic interactions (i.e. rhizosheath; sensu [49]) favour the over-representation of tolerant clades, possibly to the exclusion of non-tolerant phyla [50]. Consequently, in extreme ecosystems, the phylogenetic structures of microbial communities are expected to converge in low-diversity communities [30, 48]. In the rhizosheath-root system of the three speargrass species, a consistent microbial assembly process was observed. This was found to be largely neutral and principally driven by abiotic and biotic filtering (from desert dune conditions and the rhizosheath-root system). Plant species-related factors were found to be too weak to impose selection, minimizing the effects of differences in phylogenetic affiliation, radical exudation profiles and/or physiological status of speargrasses species [28, 31]. In a more controlled arid ecosystem, such as desert-farms, in which soil microorganisms are more abundant and diverse than the ones in barren sand [6], plants genotype were found to be important drivers of rhizospheric microbial taxonomical and functional (e.g. nitrogen fixation) diversities [3, 5].

Not surprisingly, *Actinobacteria*, *Alphaproteobacteria* and *Chloroflexi* dominated the Namib Desert bulk dune sand communities. These ubiquitous phyla have been detected in desert sand at a global scale and include members well known for their multiple genetic and physiological mechanisms of resistance to arid and oligotrophic desert conditions [51]. These include the possession of multi-stress related genes and physiological resistance mechanisms to the arid and oligotrophic desert conditions [52]. For instance: the *Chloroflexi*’s protective layered cell envelope structure [53], or sporulation of *Actinobacteria* and some *Alphaproteobacteria* [54, 55]. *Actinobacteria* and *Alphaproteobacteria* were also found to be abundant in the rhizosheath-root systems of the three speargrass species, while the relative abundance of plant-associated *Chloroflexi* was low. Notably, the most abundant actinobacterial and alphaproteobacterial taxa in the rhizosheath-root systems of the three speargrass species are known to be plant-associated bacteria with PGP potential [18, 25, 56]. *Firmicutes* were enriched only in the internal root tissues.

Fungi are well-known for high levels of stress-resistance and for their capacity to tolerate desiccation [57]. However, their roles in the rhizosheath-root system remained unclear [12, 14, 38], although it is likely that the mycelial morphology contributes to the stability of the rhizosheath structure. The rhizosheath and rhizosphere compartment of speargrass species were all enriched in fungi belonging to the *Arthoniomycetes*, *Dothideomycetes*, *Eurotiomycetes* and *Sordariomycetes* classes (all in the *Ascomycota* phylum). Among these, the most abundant genera included *Curvularia*, *Aspergillus*, *Sordaria*, *Thielavia* and *Aureobasidium*. Isolated members from these fungi groups have showed heterogeneous characteristics ranging from saprophytes to plant pathogens [58]; many of these genera also possess PGP potential (e.g., biological control of plant diseases [59]).

Intra- and inter-kingdoms interactions have previously been found to be important in shaping desert soil microbial communities [60, 61]. Bacterial and fungal components interact to form complex microbial networks in the rhizosheath-root system of the three speargrass species. In contrast, in bulk sand, disconnected micro-habitats and the presence of higher numbers of dormant cells may explain the lower complexity and the identification of co-presence interactions only [62].

Microbial hubs with high degrees of connection (up to 5%) are considered to play crucial roles within a given microbiome, and among these, the top 1% (i.e., the keystone taxa) maintain the network stability and structure [34]. Hub taxa were mainly affiliated to *Alphaproteobacteria*, with few *Actinobacteria* and *Planctomycetes.* A number of hub and keystone microbial species were specific of the three speargrasses, possibly linked to the microdiversity (SVs) of phylogenetically close taxa with conserved functional traits [63]. Only three SVs belonging to the genus *Microvirga* were identified as hubs in the microbial network of all the three plants. Members from this genus are soil bacteria which can proliferate in arid conditions [64] and provide nutrients, such as nitrogen, to plants (e.g. legume symbionts, [56]) and surrounding microbial communities (e.g. in desert soil, [65] and biological soil crusts [66]). *Microvirga* species genomes also contain genes coding for chemotaxis, motility and exopolysaccharide synthesis proteins, which facilitate movement toward and adhesion to areas of favorable nutrient conditions [66, 67], such as the rhizosheath-root systems of speargrasses. *Microvirga* are also capable of iron acquisition via siderophores [66]. Such ‘opportunistic’ interactions with the others members of the community, along with the capacity of *Microvirga* species to perform key biogeochemical processes (organic nutrient mineralization and nitrogen fixation) and to stabilize rhizosheath structures (via the production of exopolysaccharides; [66]) may explain their central role as keystone species in the microbial networks of both rhizosheath-root systems and bulk sand.

Notably, as in plant-microbe symbiotic relationships, microorganisms have evolved a structured and intimate relationship with their plant host [68] in which functional redundancy is crucial for maintaining a functioning ecosystem, especially when stresses are present [69]. In the case of speargrasses, the favourable ecological-niche created by rhizosheath-root system constitutes a refuge for microorganisms carrying biofertilization and biopromotion PGP activities (e.g., nitrogen metabolism and water retention; [11, 18, 25]) which are essential for survival in nutrient-poor arid soils.

## Conclusion

The relative simplicity of desert ecosystems, characterized by low microbial and plant diversities, allowed the evaluation of rhizosheath-root system recruitment processes and the elaboration of new general concepts in plant-microbe interactions. The present contribution provides a comprehensive study on how speargrasses species adapted to sandy desert recruit the microbial communities of their created niches (rhizosheath, rhizosphere and root) mainly from the surrounding soils. In fact, the uniqueness of rhizosheath-root system and the strong selection driven by the harsh condition of the desert ecosystems determine a stochastic (random) recruitment process conserved in all the three Namib Desert endemic plant species analysed. Experiments growing these plants in more controlled laboratory settings with different soils (e.g., oligotrophic vs rich) and with various microbial inocula (e.g., sterilized soils with different microbial inoculum complexities) will be useful to further dissect the stochasticity of the recruitment process in the speargrasses rhizosheath root systems. Even though the microbial community assembly is independent from the plant host, it yet favours the fitness of the hosts.

Our finding supports the concept that the selection determined by the low-resource condition of the desert sand prevails on that imposed by the genotype of the different plant species, suggesting that the desert microbial community assembly processes of plant-associated niches differ from those occurring in the resource-rich soils [3, 5].

Interestingly, the rhizosheath-root system has been demonstrated to be a ‘hot spot’ for microbial diversity that as previously demonstrated have the capacity to perform PGP functions and services involved in plant growth promotion (e.g. nitrogen fixation, [25]) and protection under stress conditions (e.g. exopolysaccharide production [15]). These results therefore lead to the better understanding and future modelling of plant-microbe interactions in hot and arid environments, which could be fundamental in predicting plant (including food-crop) adaptation to global climate change.

## Material and methods

### Site description, sampling and processing

In April 2017, three different species of speargrasses growing on the eastern part of a single linear dune of the Namib Desert (longitude, S 23°43′56.38″; latitude E 15°46′26.39″) were selected for this study. The plants have been identified by morphological recognition as *S. sabulicola, S. seelyae* (in literature previously defined as *S. namaquensis*) and *C. spinosa*. The selected plant species were distributed along the dune following a conserved pattern and formed a consistent ecological setting across the eastern edge of the Namib Desert (Fig. 1a; [26, 33]). For each species, the rhizosheath-root system of seven randomly selected healthy speargrasses of similar size was collected. Only mature plants with well-defined rhizosheath-root systems were sampled to minimize the potential role of developmental stage in microbial communities recruitment and assemblage [28]. After removing the sand covering the plants’ root systems, the rhizosheath-root system was sampled using sterile scissors and tweezers at 10–30 cm from the collar and placed in 50 ml sterile tubes. In addition, bulk sand samples (10–15 cm depth; *n* = 7) were collected. All the samples were collected under the research/collection permit number 2248/2017 (Namibian Ministry of Environment and Tourism).

In the laboratory, sand that was not tightly bound to the rhizosheath structure and that collected at the bottom of the tubes was transferred to 2 ml sterile tubes. Such sand was defined as belonging to the rhizosphere following the classification revised by Pang et al., [12]. The rhizosheath, which is the sandy coating physically adhering to the plant root [12], was physically separated from the inner root tissues (internal tissues) using a sterile scalpel. Samples were stored at 4 °C for soil chemical analysis and at − 20 °C for molecular analysis.

### Scanning electron microscopy (SEM) of rhizosheath-root system sections

Intact samples of rhizosheath-root systems collected from the three speargrass species were preserved and fixed in a solution of 3% glutaraldehyde in cacodylate buffer (Electron Microscopy Sciences, PA, USA) at 4 °C. Samples were rinsed three times for 15 min with a solution of 0.1 M Na-cacodylate buffer and further post-fixated in the dark for 1 h using a 1% osmium tetraoxide solution prepared with 0.1 M Na-cacodylate buffer. After post-fixation, samples were rinsed with distilled water three times for 15 min. Dehydration steps of 15 min were performed using a series of ethanol solutions of increasing concentration up to 100% (ethanol gradient: 30%, 50%, 70%, 90%, 100%). After reaching the 100% ethanol step, samples were rinsed again twice with absolute ethanol for 15 min and kept overnight in the same solution. Drying of samples was performed through evaporation of hexamethyldisilazane (HMDS) with steps of 15 min using gradually increasing concentrations of HMDS in absolute ethanol (33%, 66%, and 100% HDMS), and the last step was repeated for 1 h. When the sample was submerged in the final 100% HMDS solution, it was left loosely capped in a fume hood until all the HMDS solution had evaporated. Dried roots were attached to aluminium stubs with carbon tape and coated with a 5 nm layer of Au/Pb using a K575X sputter coater (Quorum) and visualized with a SEM Quanta 600 FEI of the KAUST Imaging and Characterization Core Lab at a working distance of 9.3 mm and a high voltage of 5.00 kV.

### Total DNA extraction

The total DNA extraction of sandy compartments (bulk sand, rhizosphere and rhizosheath) was performed using 0.5 ± 0.05 g of sample and the PowerSoil® DNA Isolation Kit (MoBio Inc., USA). For the root tissues, the surface was previously sterilized as described by Cherif et al., [22] and subsequently grinded in liquid nitrogen with sterile mortar and pestle. The total DNA extraction of the root tissues was performed using one gram of the grinded tissue and the DNeasy Plant Maxi Kit (Qiagen, Germany).

### Illumina sequencing and metaphylogenomic analysis of 16S rRNA and ITS genes

For the analysis of bacterial community composition, a PCR amplification of the V3-V4 hypervariable regions of the 16S rRNA gene was performed to the extracted DNA using universal primers (341f, 785f) as described by Mapelli et al., [62]. For fungal communities, amplification of the ITS2 region was performed using the primers ITS3f and ITS4r as described by Tedersoo et al. [70]. Both libraries were constructed with the 96 Nextera XT Index Kit (Illumina) following the manufacturer’s instructions. Library sequencing was done using the Illumina MiSeq platform with pair-end sequencing at the Bioscience Core Lab, King Abdullah University of Science and Technology. All sequenced reads were deposited in the NCBI database under the SRA accession numbers SRP153940 and SRP153934 for bacteria and fungi, respectively. Raw forward and reverse reads for each sample were assembled into paired-end reads (minimum overlap of 50 nucleotides and maximum of one mismatch within the region) using the fastq-join algorithm (https://github.com/brwnj/fastq-join) and analysed using the DADA2 pipeline as described in Callahan et al., [71]. Quality filtering, trimming, dereplication, and paired-end merging of the sequences were applied together with the final removal of sequence variants (SVs) presented in single copy and SVs classified as chloroplast (65, 8, 0.05 and 0% of sequences in root tissues, rhizosheath, rhizosphere and bulk soil, respectively). A total of 1,830,127 (average length of 405 bases) and 3,669,396 (average length of 310 bases) sequences were finally obtained for bacterial and fungal components, respectively. All samples analysed presented a suitable sequencing depth and diversity (Good’s coverage values > 98%). SVs were clustered [71] and then taxonomically assigned using the SILVA 132 database for bacteria and the UNITE database for fungi.

### Quantification of the bacterial and fungal communities by quantitative PCR (qPCR) in rhizosheath-root system compartments of speargrasses species

Absolute abundances of the number of copies of the bacterial 16S small subunit rRNA gene and the fungal ITS region were determined following the method described elsewhere, using the primer-sets Eub338/Eub518 and ITS1F/5.8 s respectively [72, 73]. For bacteria, the fragment of interest was amplified from environmental DNA (size ± 180 bp), while for fungi, it was obtained using the genomic DNA of *Saccharomyces cerevisiae* NCYC 1006 (± 450 bp). PCR products were purified with the Wizard® SV Gel and PCR Clean-Up System (Promega) and ligated to vectors pCRTM 2.1-TOPO®. The plasmids were then cloned into TOP10 *Escherichia coli* competent cells (TOPO® TA Cloning® Kit, Thermo Fischer Scientific). The plasmids, isolated from LB over-night cultures of the transformant *E. coli* using the Pure Yield Plasmid Miniprep (Promega), were used as a template to amplify the region of insertion of the fragment of interest with the primer-set M13F(-20)/M13R. PCR products were purified and then quantified using the Qubit dsDNA BR Assay Kit (Thermo Fisher Scientific). Series of standards were prepared through tenfold serial dilutions of the quantified PCR product using the Robotic workstation Qiagility (Qiagen) and stored at − 20 °C. Quantitative PCR reactions were set up with the Qiagility and were carried out on a Rotor-Gene Q thermocycler (Qiagen). All the samples were first quantified with Qubit dsDNA BR Assay Kit. Dilutions to 1 ng/μl of each sample were prepared to be used as template DNA for the qPCR runs. When the concentration of a sample was too low, such sample was used undiluted. One bulk sample was chosen as inter-run calibrator: it has been quantified in all qPCR experiments and then all the results have been normalized against it. Reaction mixes were prepared with the GoTaq® qPCR Sybr Green Master Mix (Promega). The volume of the reaction mix was 15 μl, containing 1X GoTaq® Master Mix, 100 nM of each primer for bacteria, while 400 nM for fungi, and 1.5 μl of template DNA. PCR conditions were the following: 95 °C for 2 min, 45 cycles at 95 °C for 15/40 s (respectively, bacteria/fungi), 53/55 °C for 20/40 s and 60 °C for 20/60 s; finally, melting curves were obtained through 91 cycles from 50 °C to 95 °C with increase of 0.5 °C/cycle every 5 s. Standard curves were constructed with a series of dilutions ranging from 50 to 5 × 10^7^ copies of PCR product per microliter. All the standards and the samples were run in triplicate. *R*^2^ between 0.99309 and 0.99908 and amplification efficiencies between 89% and 99% were obtained across the three different qPCR assays performed with both primer sets. To compare numbers of bacteria and fungi hosted by plant species and rhizosheath-root system compartments, the non-parametric Kruskal Wallis and post hoc Dunn’s multiple comparison tests were used.

### Microbial diversity, taxonomic distribution and statistical analyses

Bipartite network analysis was performed to the bacterial and fungal communities associated with the bulk soil, rhizosphere, rhizosheath and root tissues of the three species of speargrass using the Quantitative Insights Into Microbial Ecology (QIIME) script *make_bipartite_network.py* and visualized using the Gephi software [74]. Shared and exclusive SVs among the different compartments and speargrass species were calculated as described in Marasco et al., [75], using Venn diagram software available at http://bioinformatics.psb.ugent.be/webtools/Venn/. Ternary plots were obtained using R package (ggtern) to depict the distribution of bacterial and fungal SVs among the three different plant species [76].

Similarity matrices, Principal Coordinates Analysis (PCoA) and permutational multivariate analyses of variance (PERMANOVA, main and multiple comparison tests) have been performed on the compositional (Bray-Curtis of the log-transformed SV table) matrices in PRIMER [77]. The considered explanatory variables were ‘Plant species’ (three levels: *S. sabulicula, S. seelyae*, *C. spinosa*), ‘Compartment’ (four levels: root tissues, rhizosheath, rhizosphere, bulk sand) and their interaction (‘Compartment’ × ‘Plant species’). The occurrence of distance-decay patterns in rhizosheath-root system compartments has been tested using the linear regression (GraphPad Prism 7 software, La Jolla California USA, www.graphpad.com) between the dissimilarity of bacterial communities (Bray-Curtis) and the distance among plant species. Covariance of regressions was tested using paleontological statistics (PAST) software (one-way analysis of covariance (ANCOVA)). Alpha diversity indices (richness and evenness) were calculated using the PAST software.

To evaluate the phylogenetic community assembly, measures of phylogenetic alpha diversities (Faith’s PD, NRI and NTI) were calculated within each sample category (speargrass species and bulk sand). They were calculated for bacterial and fungal host-associated communities using the distance tree output from QIIME built including all bacterial and fungal SV, respectively [78], as well as abundance data in the R package picante [79]. Because of the autocorrelation between Faith’s PD metric and richness (bacteria: adjusted *r*^2^ = 0.94, *r* confidential interval 0.96 to 0.98, *p* < 0.0001; fungi: adjusted *r*^2^ = 0.91, *r* confidential interval 0.92 to 0.97, *p* < 0.0001), the ratio PD/SV has been used to investigate the difference explained by phylogenetic diversity excluding the possible artefact due to abundance counts. NRI and NTI examined whether co-occurring taxa are closely related than expected by chance, providing information at deep-level relatedness and finer-scale of phylogeny, respectively [80]. Positive values of NTI and NRI (> 0) indicate phylogenetic clustering (i.e., SVs within the host are more closely related than expected by chance), whereas negative values (< 0) indicate phylogenetic overdispersion (i.e., SVs within the host are less closely related than expected by chance; [80]). Estimation of phylogenetic turnover (ßNRI and ßNTI) has been conducted using the function ‘comdistnt’ in R with ‘picante’ package [81]. βNRI values <− 2 indicate significantly less than expected phylogenetic turnover (homogeneous selection), whereas βNRI values > + 2 indicate significantly more than expected phylogenetic turnover (variable selection; [31]). Differences in mean phylogenetic alpha and beta diversities between the hosts were assessed with ANOVA, and post hoc pairwise comparisons (Newman-Keuls Multiple Comparison Test) were performed in GraphPad Prism 7 software. Kruskal-Wallis test (FDR p correction) was used to detect the difference among taxonomic groups in rhizosheath-root system compartments and species.

### Co-occurrence network analysis

Co-occurrence relationships were analysed for each plant species and bulk soil using the CoNet plugin of Cytoscape 3.4 and visualized using Gephi 0.9.1 [74]. A combination of the Bray-Curtis (BC) and Kullback-Leiber (KLD) dissimilarity indices, along with the Pearson and Spearman correlation coefficients, were used to build the network. Edge-specific permutation and bootstrap score distributions with 1000 iterations were performed. The obtained data was normalized to detect statistically significant non-random events of co-occurrence (co-presence and mutual exclusion). The *p* value was computed by *z*-scoring the permuted null and bootstrap confidence interval using pooled variance [82]. The most important statistical network descriptors were calculated [83]. Node centralization descriptors such as degree, betweenness centrality, closeness centrality and average shortest path length were normalized using a standardization method (n1) for visualization purposes. The effect of compartment and plant species was assessed for the three main node centrality parameters: (i) generalized linear model with a quasi-binomial distribution of error was performed for betweenness centrality; (ii) ANOVA of log-transformed values was used for closeness centrality; (iii). ANOVA on normally distributed values was applied for the average path length. Hubs and keystone species were identified separately for each speargrass and bulk sand networks. Hubs were defined as those nodes within the top 5% of degree values in a network, while keystones were defined considering the top 1%.

## Additional file

Additional file 1: **Table S1.** Soil physico-chemistry of the dune’s bulk sand. All values are given as mean of three replicates ±  standard error. **Table S2.** Measurements of root and rhizosheath diameters (*n* = 10). Analysis of variance (ANOVA) is reported. For values *p* < 0.005 post-hoc comparison (Tukey’ test) was done, letters in parenthesis indicate the results of multiple comparisons. **Table S3.** Results of ANOVA multiple comparison tests analyzing the intraspecific dissimilarity associated to the hosts and bulk sand were reported for (a) bacterial and (b) fungal communities. Average distance from centroid was used as measure of dispersion. Significant differences (*p* < 0.05) among pair host (speargrasses and bulk sand) were indicated with star (*). **Table S4.** (a) Estimation of components of variation in bacterial and fungal communities. (b and c) Multi comparison tests (PERMANOVA, number of permutation = 999) for bacterial and fungi, respectively, considering plant species or rhizosheath-root compartments. (d) Mantel test results showing correlations between compositional beta diversity associated to compartments and distance from the dune bottom for both bacteria and fungi. Significance *p* < 0.05 **Table S5.** (a) Covariance (ANCOVA) and (b) linear regression analysis of distance decay rates for compositional (Bray-Curtis) similarity in the rhizosheath-root system compartment. Results were reported for bacterial and fungal components. **Table S6.** Mantel test results showing correlations between phylogenetic alpha-diversity metrics associated to compartments and distance from the dune bottom for both bacteria and fungi. **Table S7.** Taxonomical classification of (a) bacteria and (b) fungi with relative abundance expressed in percentage. See excel file named Additional file 1: Table S7. **Table S8.** Evaluation of the effect of single factors ‘Plant species’ and ‘Compartment’ and their interaction (Plant species ´ Compartment) on bacterial and fungal taxonomical distribution using PERMANOVA (main test). Taxonomical distribution have been analyzed at phylum/class and family level for bacteria (99 and 82% sequences classified; a and b, respectively) and at class and genus level for fungi (85 and 70% sequences classified; c and d, respectively). Significant PERMANOVA results (*p* < 0.05) were indicated with star (*). **Table S9.** Kruskal-Wallis test to evaluate significant differences in (a) bacterial and (b) fungal relative abundance across groups. See excel file named Additional file 1: Table S9. **Table S10.** Network table with list of nodes and edge. See excel file named Additional file 1: Table S10. **Figure S1.** Venn diagram detecting percentage of bacterial and fungal SVs shared among the rhizosheath-root system compartments (root, rhizosheath and rhizosphere) and bulk sand of all the three species studied. Biggest numbers indicate the percentage of SVs and the numbers in parenthesis the relative abundance of those SVs. **Figure S2.** Venn diagram detecting percentage of bacterial (upper panels) and fungal (lover panels) SVs shared among the rhizosheath-root system compartments (root, rhizosheath and rhizosphere) and bulk sand for each of the three species studied. Biggest numbers indicate the percentage of SVs and the numbers in parenthesis the relative abundance of those SVs. **Figure S3.** Venn diagram detecting bacterial (upper panels) and fungal (lover panels) SVs shared among the three speargrasses species (*S. sabulicola*, *S. seelyae* and *C. spinosa*) for each rhizosheath-root system compartments (root, rhizosheath and rhizosphere). Biggest numbers indicate the percentage of SV and the numbers in parenthesis the relative abundance of those SVs. **Figure S4.** Analysis of edge betweenness centrality in speargrasses rhizosheath-root system networks. Color code indicated the interaction among different pair of phylogenetic group. Name of the phylogenetic group are reported in the vertical axis. (ZIP 3591 kb)

## Abbreviations

ANCOVA: Analysis of covariance
BC: Bray-Curtis
CPM: Counts per million
FDR: False discovery rate
HMDS: Hexamethyldisilazane
ITS: Internal transcribed spacers
KLD: Kullback-Leiber
NRI: Relatedness index
NTI: Nearest taxon index
PAST: Paleontological statistics
PCoA: Principal coordinate analysis
PCR: Polymerase chain reaction
PD: Phylogenetic distance
PERMANOVA: Permutational multivariate analysis of variance
PGP: Plant growth-promoting
QIIME: Quantitative Insights Into Microbial Ecology
qPCR: Quantitative polymerase chain reaction
rRNA: Ribosomal RNA
SD: Standard deviation
SEM: Scanning electron microscope
ßNRI: Basal phylogenetic beta diversity
ßNTI: Terminal phylogenetic beta diversity
SV: Sequence variant
