# Supplementary material for: Rhizosheath microbial community assembly of sympatric desert speargrasses is independent of the plant host
Source: Microbiome. 2018 Dec 4;6:215. doi: 10.1186/s40168-018-0597-y (PMC6280439; doi:10.1186/s40168-018-0597-y)

**ADDITIONAL INFORMATION**

**Desert speargrass rhizosheath microbial community assembly is stochastic and independent of plant host phylogeny**

Ramona Marasco^1#*^, María J. Mosqueira^1#^, Marco Fusi^1^, Jean-Baptiste Ramond^2^, Giuseppe Merlino^1^, Jenny M Booth^1^, Gillian Maggs-Kölling^3^, Don A Cowan^2^, Daniele Daffonchio^1*^

^1^ King Abdullah University of Science and Technology (KAUST), Biological and Environmental Sciences and Engineering Division (BESE), Thuwal 23955-6900, Saudi Arabia

^2^ University of Pretoria, Department of Biochemistry, Genetics and Microbiology, Centre for Microbial Ecology and Genomics, Pretoria, South Africa

^3^ Gobabeb Research and Training Centre, Walvis Bay, Namibia

^#^ Equal contribution

^*^ Authors for correspondence: Daniele Daffonchio, King Abdullah University of Science and Technology (KAUST), Biological and Environmental Sciences and Engineering Division (BESE), Thuwal 23955-6900, Saudi Arabia. Phone: +966 (0)12 8082884; E-mail: [daniele.daffonchio@kaust.edu.sa](mailto:daniele.daffonchio@kaust.edu.sa); Ramona Marasco, King Abdullah University of Science and Technology (KAUST), Biological and Environmental Sciences and Engineering Division (BESE), Thuwal 23955-6900, Saudi Arabia. E-mail: ramona.marasco@kaust.edu.sa

**ADDITIONAL TABLES**

**Additional file Table S1**. Soil physico-chemistry of the dune’s bulk sand. All values are given as mean of three replicates ± standard error.

| **Variable measured** | | **Average (n=3)** | **St.Er.** |
| --- | --- | --- | --- |
| Soil  Chemistry | Ph | 7.43 | 0.15 |
|  | Exchange capacity (mS/m) | 12.34 | 1.00 |
|  | Organic carbon (%) | 0.01 | 0.02 |
| Nutrients  (mg/L) | Phosphorous | 0.24 | 0.02 |
|  | Sodium | 4.30 | 0.24 |
|  | Potassium | 6.40 | 0.59 |
|  | Calcium | 17.73 | 1.22 |
|  | Magnesium | 3.79 | 0.16 |
|  | Chlorine | 16.85 | 0.76 |
|  | Sulfate | 4.90 | 1.47 |
|  | Ammonium | 0.56 | 0.31 |
|  | Nitrate | 1.93 | 0.64 |
| Granulometry (%) | Gravel (> 5mm) | 0.00 | 0.00 |
|  | Small gravel (> 2mm) | 0.00 | 0.00 |
|  | Coarse sand (>1000 µm) | 0.32 | 0.43 |
|  | Coarse sand (>500 µm) | 0.25 | 0.12 |
|  | Medium sand (> 250 µm) | 64.55 | 9.28 |
|  | Fine sand (> 100 µm) | 34.00 | 8.86 |
|  | Very fine sand (> 53 µm) | 0.47 | 0.36 |
|  | Clay (< 53 µm) | 0.00 | 0.00 |
|  | Silt (< 53 µm) | 0.41 | 0.40 |

**Additional file Table S2.** Measurements of root and rhizosheath diameters (n=10). Analysis of variance (ANOVA) is reported. For values *p*<0.005 post-hoc comparison (Tukey’ test) was done, letters in parenthesis indicate the results of multiple comparisons.

| **Species** | **Root tissue (mm)** | **Rhizosheath (mm)** |
| --- | --- | --- |
| *S. sabulicula* | 3.29 ± 0.41 (a) | 1.25 ± 0.28 (a) |
| *S. selyae* | 2.79 ± 0.35 (b) | 1.01 ± 0.19 (a) |
| *C. spinosa* | 2.55 ± 0.5 (c) | 0.63 ± 0.09 (a) |

**Additional file Table S3.** Results of ANOVA multiple comparison tests analyzing the intraspecific dissimilarity associated to the hosts and bulk sand were reported for (**a**) bacterial and (**b**) fungal communities. Average distance from centroid was used as measure of dispersion. Significant differences (*p*<0.05) among pair host (speargrasses and bulk sand) were indicated with star (*).

(**a**) Bacterial component

| **Pair-comparison of host** | ***p* adjusted** |
| --- | --- |
| *C. spinose,* Bulk sand | 0.0031299* |
| *S. sabulicola*, Bulk sand | 0.0019199* |
| *S. seelyae*, Bulk sand | 0.00004* |
| *S. sabulicola, C. spinose* | 0.9963341 |
| *S. seelyae, C. spinose* | 0.2868722 |
| *S. seelyae, S. sabulicola* | 0.4006874 |

(**b**) Fungal component

| **Pair-comparison of host** | ***p* adjusted** |
| --- | --- |
| *C. spinose,* Bulk sand | 0.0056713* |
| *S. sabulicola*, Bulk sand | 0.1266092 |
| *S. seelyae*, Bulk sand | 0.0189061* |
| *S. sabulicola, C. spinosa* | 0.4257006 |
| *S. seelyae, C. spinose* | 0.948851 |
| *S. seelyae, S. sabulicola* | 0.7528356 |

**Additional file Table S4.** (**a**) Estimation of components of variation in bacterial and fungal communities. (**b** and **c**) Multi comparison tests (PERMANOVA, number of permutation=999) for bacterial and fungi, respectively, considering plant species or rhizosheath-root compartments. (**d**) Mantel test results showing correlations between compositional beta diversity associated to compartments and distance from the dune bottom for both bacteria and fungi. Significance *p*<0.05

(**a**) Estimates of components of variation. Values are expresses as percentage.

| **Kingdom** | **Factor** | **Estimate** | **Sq.root** | **Components of variation (%)** |
| --- | --- | --- | --- | --- |
| Bacteria | Plant | 254.89 | 15.965 | 6. |
|  | Compartment | 1726.1 | 41.546 | 43 |
|  | Plant × Compartment | 275.99 | 16.613 | 7 |
|  | Residual | 1761.2 | 41.967 | 44 |
| Fungi | Plant | 513.61 | 22.663 | 26 |
|  | Compartment | 45.989 | 6.7815 | 2 |
|  | Plant × Compartment | 22.667 | 4.7609 | 1 |
|  | Residual | 1360 | 36.878 | 70 |

(**b**) Bacterial component

| **Compartment factor** | ***S. sabulicola*** | ***S. seelyae*** | ***C. spinose*** |
| --- | --- | --- | --- |
| Root, Rhizosheath | t=2.950, P=0.001 | t=3.415, P=0.001 | t=3.542, P=0.001 |
| Root, Rhizosphere | t=2.935, P=0.001 | t=3.619, P=0.001 | t=3.700, P=0.001 |
| Rhizosheath, Rhizosphere | t=0.955, P=0.473 | t=1.706, P=0.007 | t=1.524, P=0.03 |
|  |  |  |  |
| **Species factor** | **Root** | **Rhizosheath** | **Rhizosphere** |
| *S. sabulicola, S. seelyae* | t=1.152, P=0.259 | t=1.575, P=0.028 | t=1.800, P=0.012 |
| *S. sabulicola, C. spinose* | t=1.909, P=0.009 | t=1.544, P=0.035 | t=1.539, P=0.038 |
| *S. seelyae, C. spinose* | t=1.821, P=0.002 | t=1.751, P=0.004 | t=1.647, P=0.004 |

(**c**) Fungal component

| **Compartment factor** | ***S. sabulicola*** | ***S. seelyae*** | ***C. spinose*** |
| --- | --- | --- | --- |
| Rhizosheath, Rhizosphere | t=0.99, P=0.389 | t=1.09, P=0.268 | t=1.18, P= 0.198 |
|  |  |  |  |
| **Species factor** | **Rhizosheath** | **Rhizosphere** |  |
| *S. sabulicola, S. seelyae* | t=1.99, P=0.01 | t=1.95, P=0.04 |  |
| *S. sabulicola, C. Spinosa* | t=1.86, P=0.01 | t=1.77, P=0.012 |  |
| *S. seelyae, C. Spinosa* | t=1.87, P=0.005 | t=1.89, P=0.005 |  |

(**d**) Mantel test correlation of bacterial and fungi components with distance from dune bottom

| **Microbe** | **Compartment** | **Compositional (Bray-Curtis)** |
| --- | --- | --- |
| Bacteria | Root | z=8007.86; *p*=0.103 |
|  | Rhizosheath | z=13550.59; *p*=0.001 |
|  | Rhizosphere | z=13749.33; *p*=0.001 |
| Fungi | Rhizosheath | z=13511.58; *p*=0.001 |
|  | Rhizosphere | z=13235.62; *p*=0.003 |

**Additional file Table S5.** (a) Covariance (ANCOVA) and (b) linear regression analysis of distance decay rates for compositional (Bray-Curtis) similarity in the rhizosheath-root system compartment. Results were reported for bacterial and fungal components.

(a) Covariance analysis

| **Microbe** | **Covariance analysis** | **Bray-Curtis** |
| --- | --- | --- |
| Bacteria | Root, Rhizosheath | F_1,397_=231, p<0.0001 |
|  | Root, Rhizosphere | F_1,397_=241, p<0.0001 |
|  | Rhizosheath, Rhizosphere | F_1,417_=2.49, p=0.12 |
| Fungi | Rhizosheath, Rhizosphere | F_1,417_=0.001, p=0.97 |

(b) Linear regression analysis

| **Microbe** | **Compartment** | **N.** | **Slope** | **95% confidence intervals** | **F** | ***p*** | **R^2^** |
| --- | --- | --- | --- | --- | --- | --- | --- |
| Bacteria | Root | 190 | -1.44 | -2.802 to -0.07518 | 4.277 | 0.04 | 0.022 |
|  | Rhizosheath | 210 | -1.72 | -2.443 to -1.000 | 21.89 | <0.0001 | 0.095 |
|  | Rhizosphere | 210 | -2.95 | -3.809 to -2.099 | 45.82 | <0.0001 | 0.180 |
| Fungi | Rhizosheath | 210 | -3.836 | -4.954 to -2.717 | 45.18 | <0.0001 | 0.178 |
|  | Rhizosphere | 210 | -3.663 | -4.592 to -2.734 | 59.70 | <0.0001 | 0.223 |

**Additional file Table S6.** Mantel test results showing correlations between phylogenetic alpha-diversity metrics associated to compartments and distance from the dune bottom for both bacteria and fungi.

| **Kingdom** | **Metric** | **Z** | **P** |
| --- | --- | --- | --- |
| Bacteria | PD/SV | 103.0245 | 0.001 |
|  | NRI | 11456.88 | 0.613 |
|  | NTI | 7183.787 | 0.967 |
| Fungi | PD/SV | 150.1399 | 0.315 |
|  | NRI | 2142.388 | 0.377 |
|  | NTI | 1976.205 | 0.833 |

**Additional file Table S7.** Taxonomical classification of (**a**) bacteria and (**b**) fungi with relative abundance expressed in percentage. See excel file named Additional file Table S7.xlsx

**Additional file Table S8.** Evaluation of the effect of single factors ‘Plant species’ and ‘Compartment’ and their interaction (Plant species × Compartment) on bacterial and fungal taxonomical distribution using PERMANOVA (main test). Taxonomical distribution have been analyzed at phylum/class and family level for bacteria (99 and 82% sequences classified; **a** and **b**, respectively) and at class and genus level for fungi (85 and 70% sequences classified; **c** and **d**, respectively). Significant PERMANOVA results (*p*<0.05) were indicated with star (*).

| **(a) Phylum/Class level – Bacteria** | **Df** | **Pseudo-F** | **P(MC)** |
| --- | --- | --- | --- |
| Plant species | 2 | 69.478 | 0.001 |
| Compartment | 2 | 1.785 | 0.094 |
| Plant species X Compartment | 4 | 2.092 | 0.026* |
| Res | 53 |  |  |
|  |  |  |  |
| **(b) Family level – Bacteria** | **Df** | **Pseudo-F** | **P(MC)** |
| Plant species | 2 | 51.371 | 0.001 |
| Compartment | 2 | 3.719 | 0.001 |
| Plant species X Compartment | 4 | 2.651 | 0.001* |
| Res | 53 |  |  |
|  |  |  |  |
| **(c) Class level – Fungi** | **Df** | **Pseudo-F** | **P(MC)** |
| Plant species | 2 | 2.5505 | 0.02* |
| Compartment | 1 | 1.5498 | 0.209 |
| Plant species X Compartment | 2 | 1.042 | 0.396 |
| Res | 36 |  |  |
|  |  |  |  |
| **(d) Genus level – Fungi** | **Df** | **Pseudo-F** | **P(MC)** |
| Plant species | 2 | 6.0054 | 0.001* |
| Compartment | 1 | 2.6376 | 0.008* |
| Plant species X Compartment | 2 | 1.1073 | 0.327 |
| Res | 36 |  |  |

**Additional file Table S9.** Kruskal-Wallis test to evaluate significant differences in (**a**) bacterial and (**b**) fungal relative abundance across groups. See excel file named Additional file Table S9.xlsx

**Additional file Table S10.** Network table with list of nodes and edge. See excel file named Additional file Table S10.xlsx

**ADDITIONAL FIGURES**

**Additional file Figure S1.** Venn diagram detecting percentage of bacterial and fungal SVs shared among the rhizosheath-root system compartments (root, rhizosheath and rhizosphere) and bulk sand of all the three species studied. Biggest numbers indicate the percentage of SVs and the numbers in parenthesis the relative abundance of those SVs.


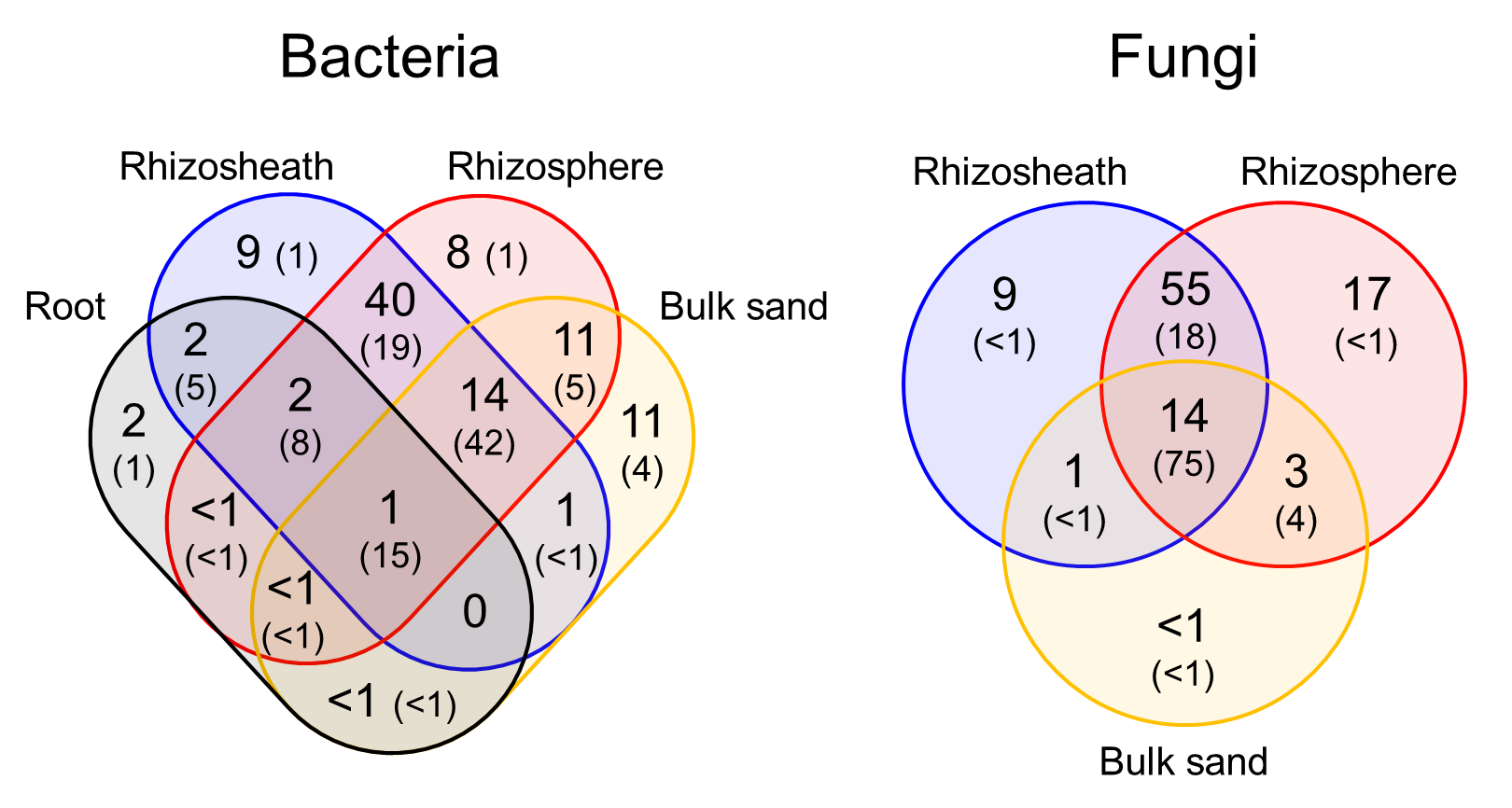


**Additional file Figure S2.** Venn diagram detecting percentage of bacterial (upper panels) and fungal (lover panels) SVs shared among the rhizosheath-root system compartments (root, rhizosheath and rhizosphere) and bulk sand for each of the three species studied. Biggest numbers indicate the percentage of SVs and the numbers in parenthesis the relative abundance of those SVs.


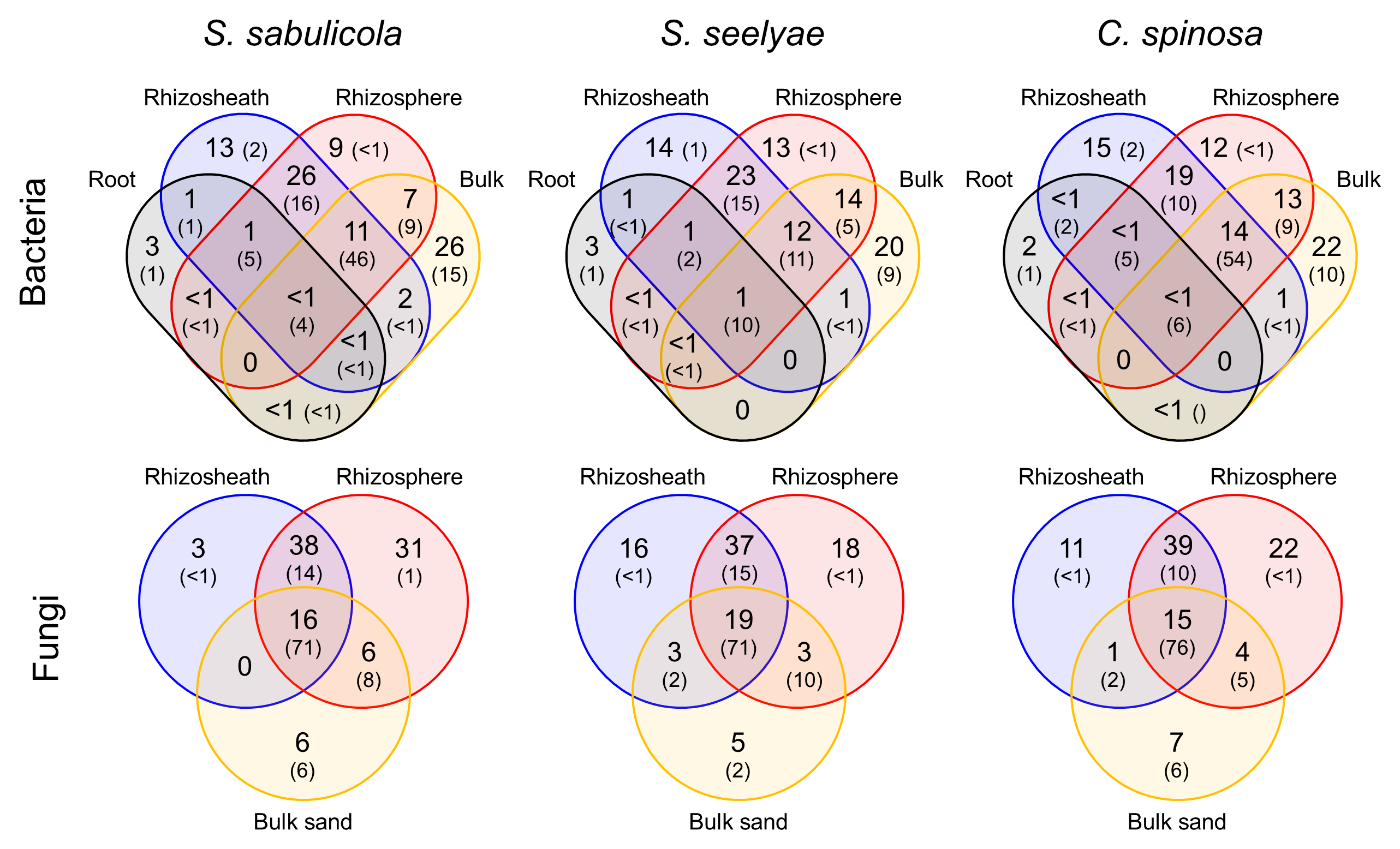


**Additional file Figure S3.** Venn diagram detecting bacterial (upper panels) and fungal (lover panels) SVs shared among the three speargrasses species (*S. sabulicola, S. seelyae* and *C. spinosa*) for each rhizosheath-root system compartments (root, rhizosheath and rhizosphere). Biggest numbers indicate the percentage of SV and the numbers in parenthesis the relative abundance of those SVs.


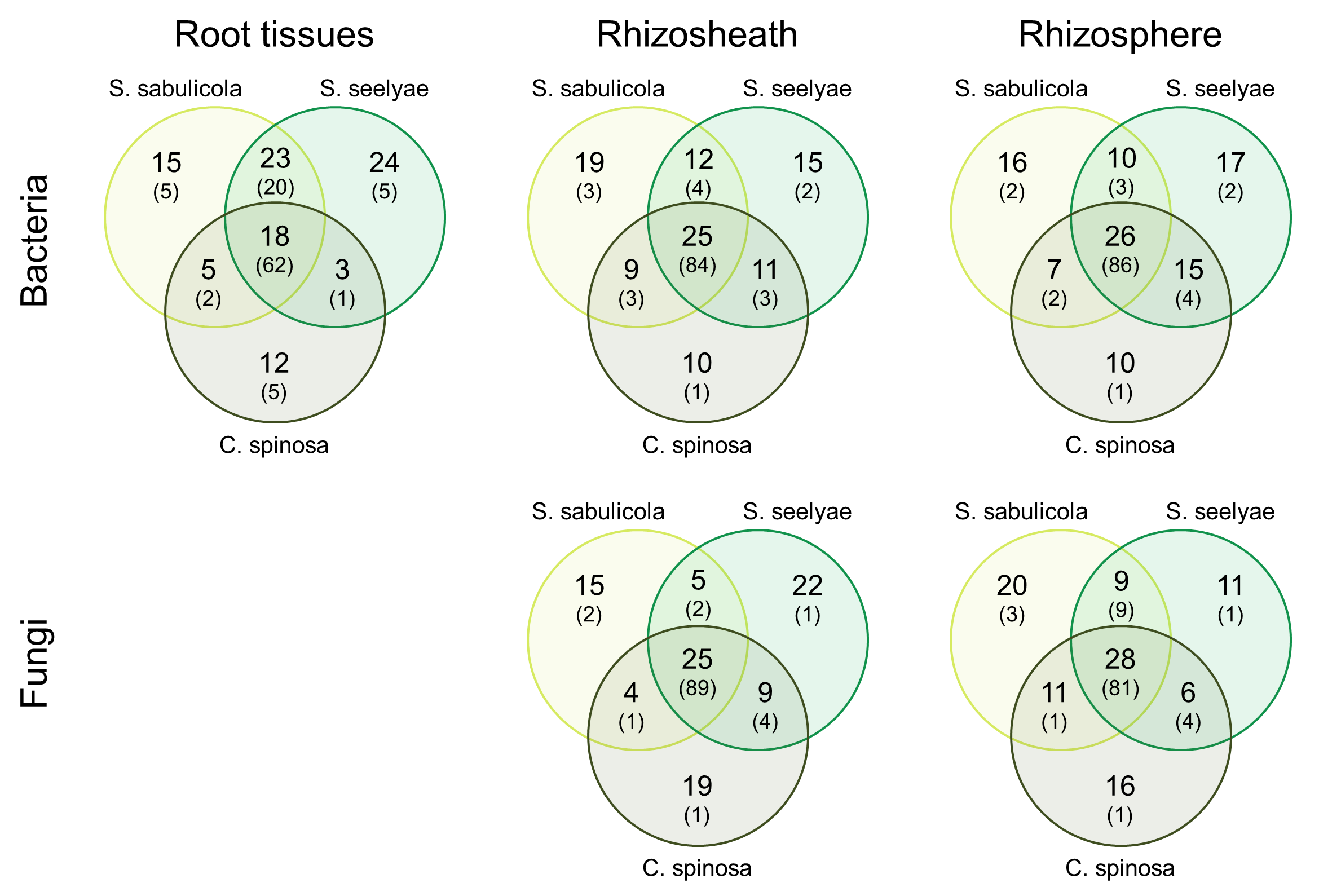


**Additional file Figure S4.** Analysis of edge betweenness centrality in speargrasses rhizosheath-root system networks. Color code indicated the interaction among different pair of phylogenetic group. Name of the phylogenetic group are reported in the vertical axis.


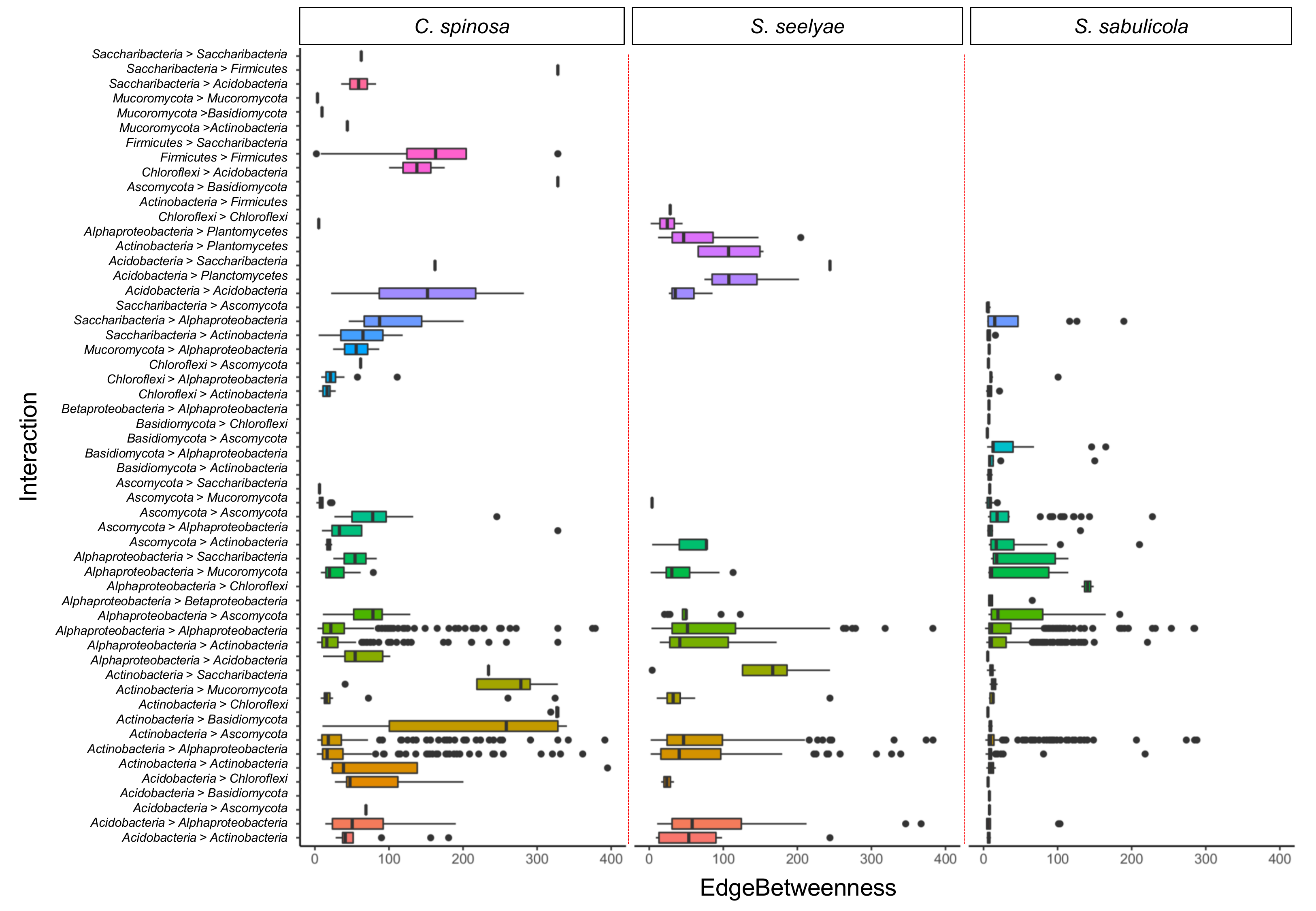

Supplement: Supplementary file 1 — Table S1. Soil physico-chemistry of the dune’s bulk sand. All values are given as mean of three replicates ± standard error. Table S2. Measurements of root and rhizosheath diameters (n = 10). Analysis of variance (ANOVA) is reported. For values p < 0.005 post-hoc comparison (Tukey’ test) was done, letters in parenthesis indicate the results of multiple comparisons. Table S3. Results of ANOVA multiple comparison tests analyzing the intraspecific dissimilarity associated to the hosts and bulk sand were reported for (a) bacterial and (b) fungal communities. Average distance from centroid was used as measure of dispersion. Significant differences (p < 0.05) among pair host (speargrasses and bulk sand) were indicated with star (*). Table S4. (a) Estimation of components of variation in bacterial and fungal communities. (b and c) Multi comparison tests (PERMANOVA, number of permutation = 999) for bacterial and fungi, respectively, considering plant species or rhizosheath-root compartments. (d) Mantel test results showing correlations between compositional beta diversity associated to compartments and distance from the dune bottom for both bacteria and fungi. Significance p < 0.05 Table S5. (a) Covariance (ANCOVA) and (b) linear regression analysis of distance decay rates for compositional (Bray-Curtis) similarity in the rhizosheath-root system compartment. Results were reported for bacterial and fungal components. Table S6. Mantel test results showing correlations between phylogenetic alpha-diversity metrics associated to compartments and distance from the dune bottom for both bacteria and fungi. Table S7. Taxonomical classification of (a) bacteria and (b) fungi with relative abundance expressed in percentage. See excel file named Additional file 1: Table S7. Table S8. Evaluation of the effect of single factors ‘Plant species’ and ‘Compartment’ and their interaction (Plant species ´ Compartment) on bacterial and fungal taxonomical distribution using PERMANOVA (main test). [file 40168_2018_597_MOESM1_ESM.zip › Marasco et al 2018 AF.docx]
